# Supplementary material for: Unraveling the influence of non-fullerene acceptor molecular packing on photovoltaic performance of organic solar cells
Source: Nat Commun. 2020 Nov 26;11:6005. doi: 10.1038/s41467-020-19853-z (PMC7693324; doi:10.1038/s41467-020-19853-z)
Supplement: Supplementary file 1 — Supplementary Information [file 41467_2020_19853_MOESM1_ESM.pdf]

SUPPLEMENTARY INFORMATION

Unraveling the influence of non-fullerene acceptor molecular packing on photovoltaic performance of organic solar cells

Ye et al.

Supplementary Figures

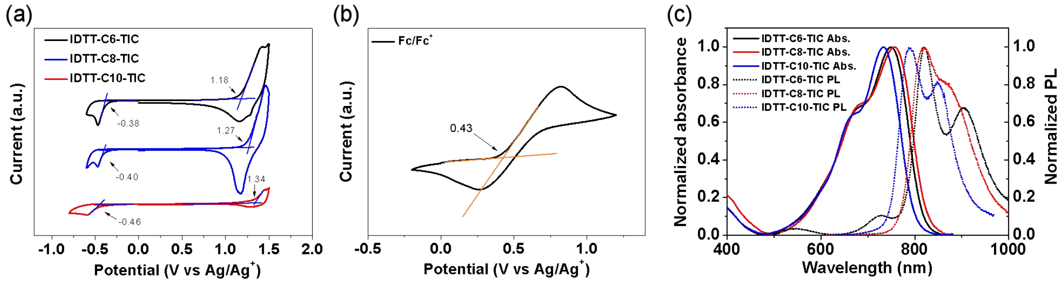

Supplementary Figure 1. Cyclic voltammetry curves of **a** IDTT-CX-TIC and **b** Ferrocene. **c** Normalized thin-film absorption and photoluminescence spectra of IDTT-CX-TIC.

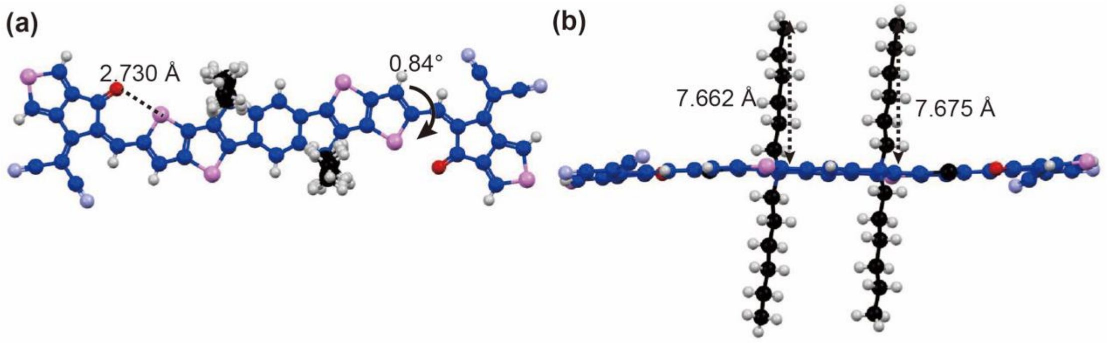

Supplementary Figure 2. a Top view, and **b** side view of molecular conformer of IDTT-C6-TIC. IDTT-C6-TIC backbone is a symmetry structure with a small torsion angle, and the O-S intramolecular interactions maintain a planar structure. The side chain C atoms are labeled as the black color. It can be seen that the side chains have a fully extended linear shape and the vertical length is around 7.6 Å. All the structure pictures are picked up from single crystal information file and draw by Mercury software.

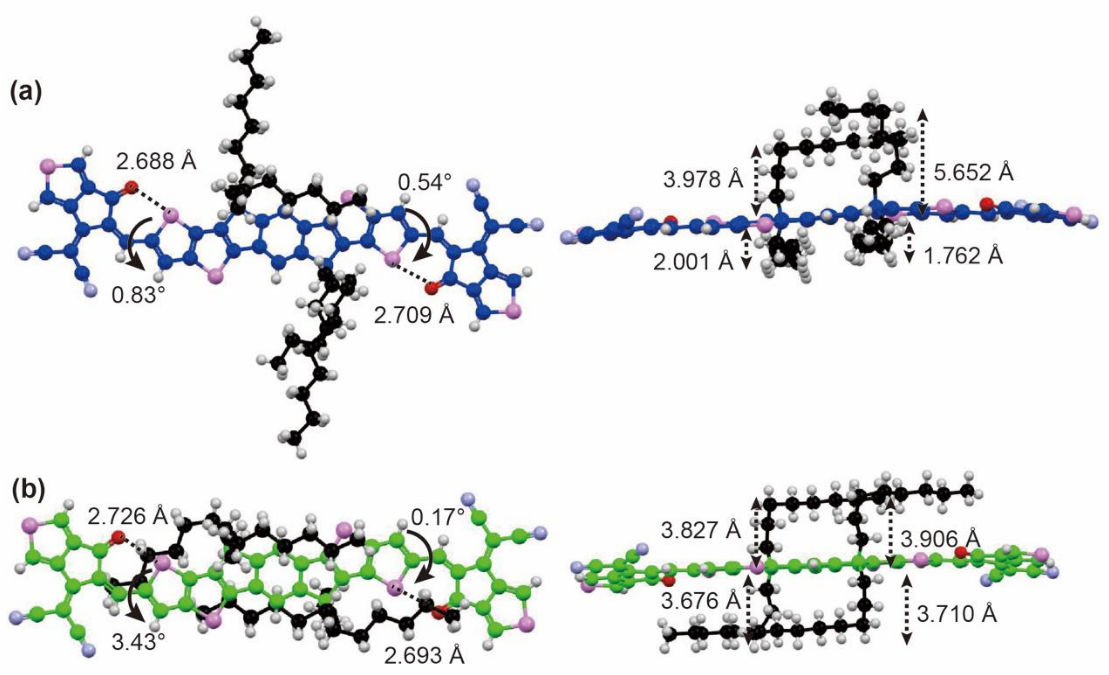

Supplementary Figure 3. a, b Two molecular conformers of IDTT-C8-TIC. Left column is the top view, and right column is the side view. Different from IDTT-C6-TIC backbone symmetry characteristics, the two IDTT-C8-TIC conformer backbones show asymmetry shape as the two molecular end-groups have different torsional angles.

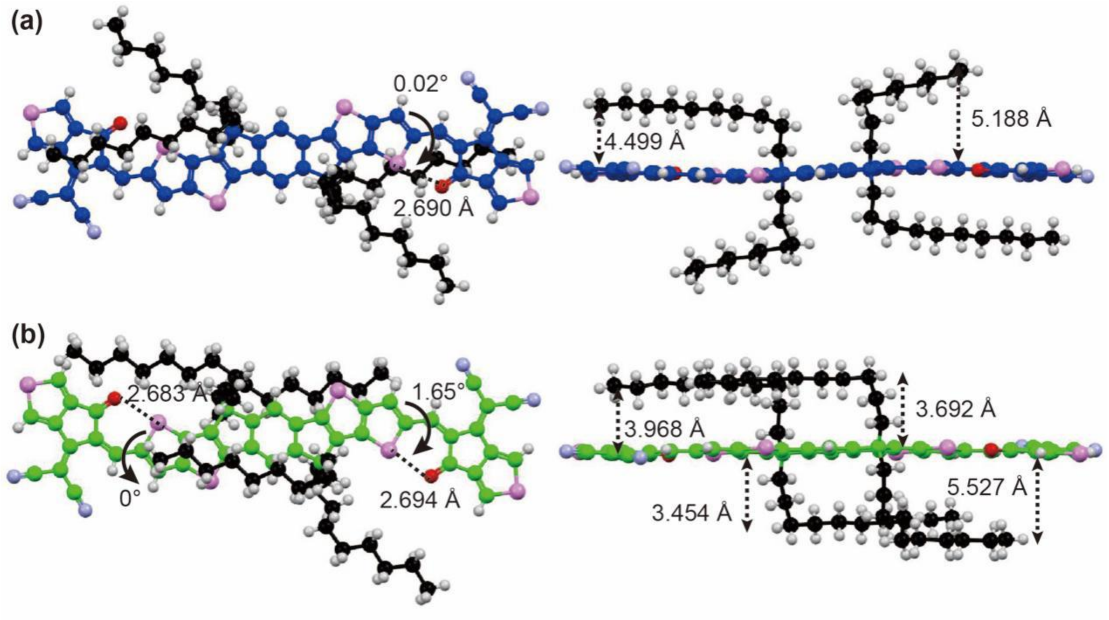

Supplementary Figure 4. Two molecular conformers of IDTT-C10-TIC. Left column is thr top view, and right column is the side view. IDTT-C10-TIC shows one symmetric backbone conformer **a** and an asymmetric backbone conformer **b**. The side chain shape leads to **a** harpoon-type and **b** crankshaft-type side chain extensions.

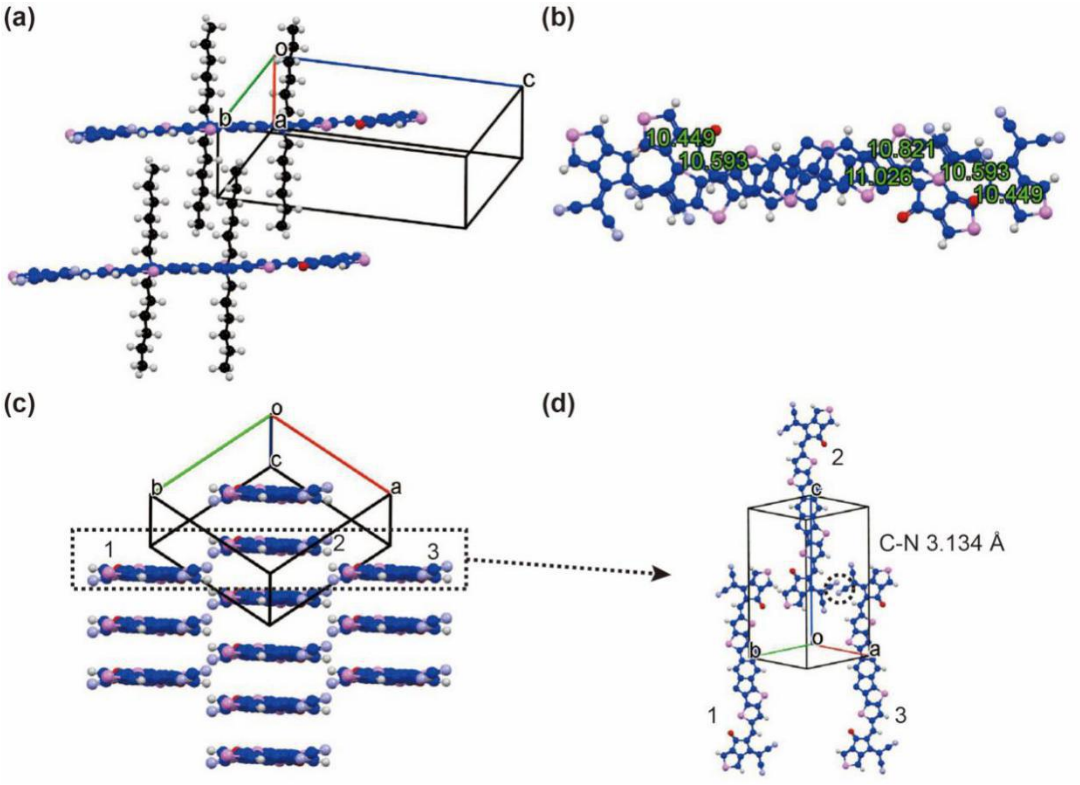

Supplementary Figure 5. a Two IDTT-C6-TIC molecules separated by the side chains, and **b** the corresponding backbone close atom-atom distances. **c** The side view from one-dimensional π-π stacking chain and **d** the corresponding close distance of the dash cube in Supplementary Figure 5c. From the top view, the CN group of molecule 2 has a small distance with molecule 3.

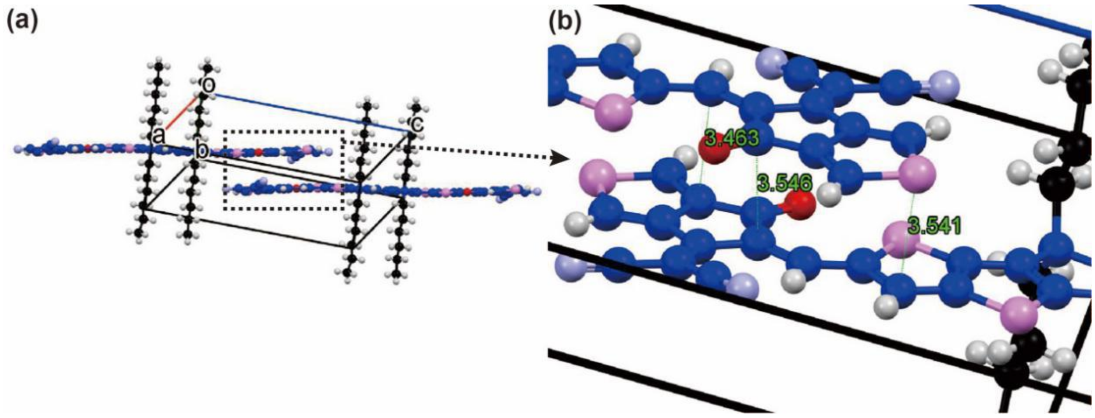

Supplementary Figure 6. a IDTT-C6-TIC molecular π-π stacking motif. **b** The atom-atom distances from two molecular end-groups.

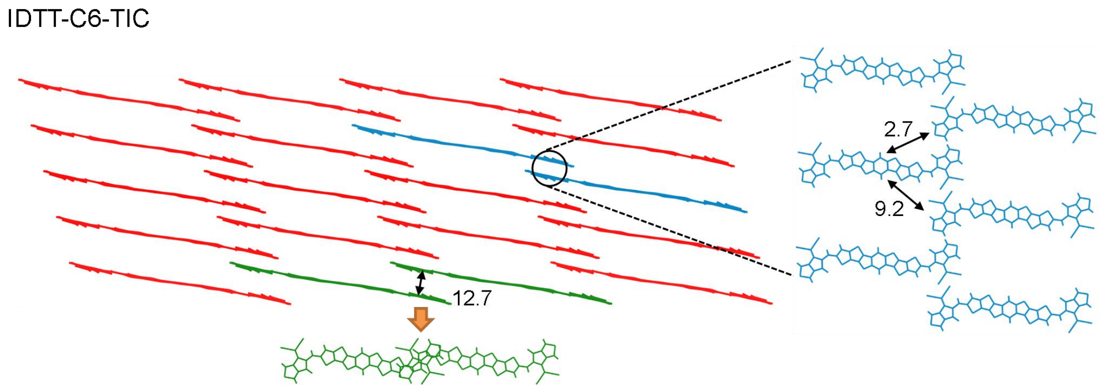

Supplementary Figure 7. Crystal packing and electronic coupling for IDTT-C6-TIC. From single crystal structure, the IDTT-C6-TIC molecule can from π - π stacking through the end group, which the transfer integral has been calculated around 12.7 meV. The CN-CN part has 9.2 meV coupling, while the TIC end-group also has 2.7 meV coupling with a neighbor backbone middle part.

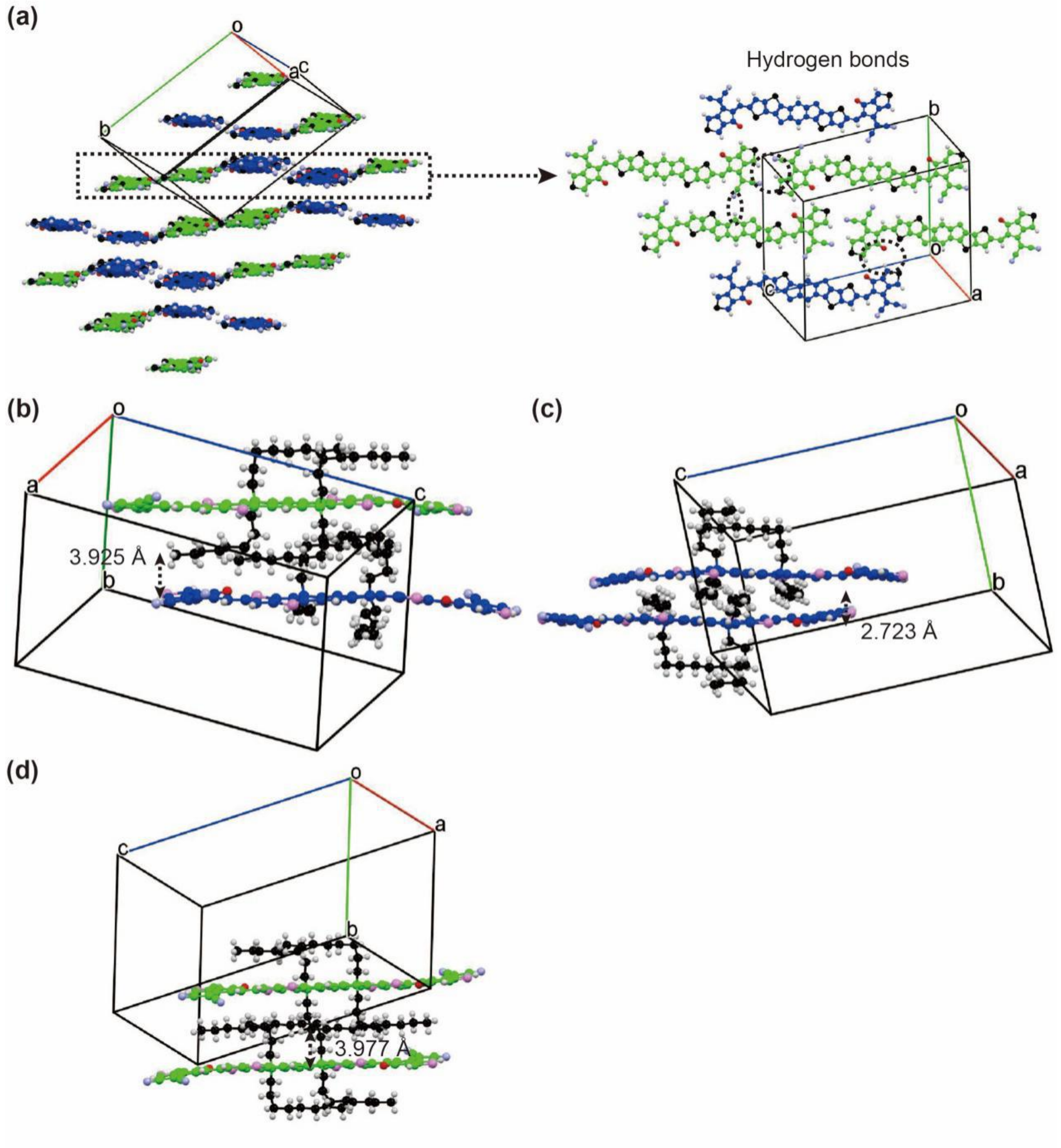

Supplementary Figure 8. a IDTT-C8-TIC molecular stacking (left), the blue and green conformers form close interactions throug hydrogen bonding (right). **b-d** Three pairs interactions in IDTT-C8-TIC.

IDTT-C8-TIC

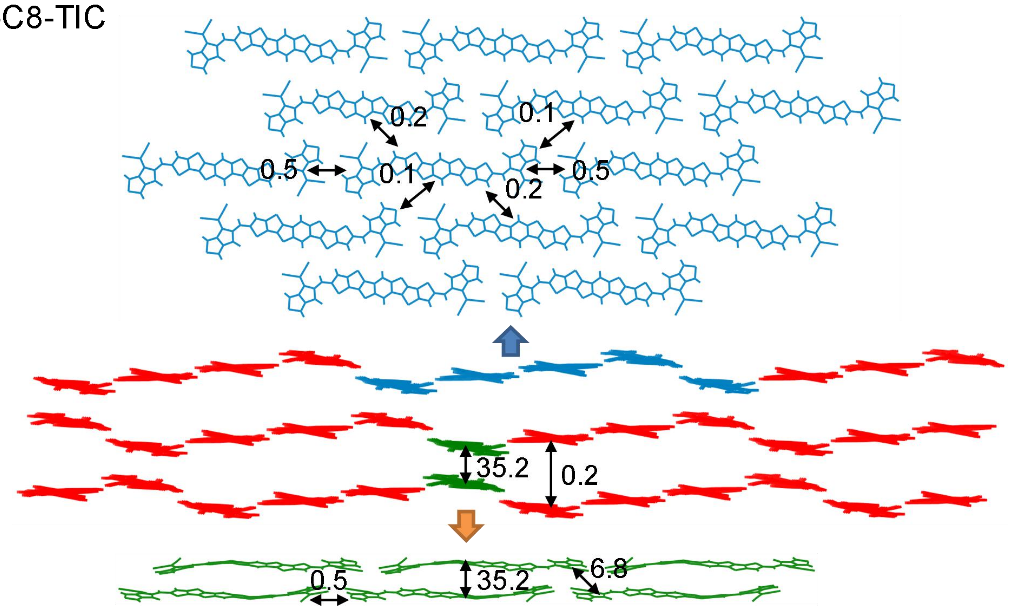

Supplementary Figure 9. Crystal packing and electronic coupling for IDTT-C8-TIC. Three pairs interactions can be introduced in IDTT-C8-TIC. The green-green dimer has a close packing distance and a large transfer integral of ~ 35.2 meV. Meanwhile, the orange-orange dimer has a very low value of ~ 0.2 meV. We also noticed that the hydrogen bonds have been found in the same altitude molecular layer. Through quantum chemistry calculation, the transfer integral values are in the range of 0.1-0.5 meV.

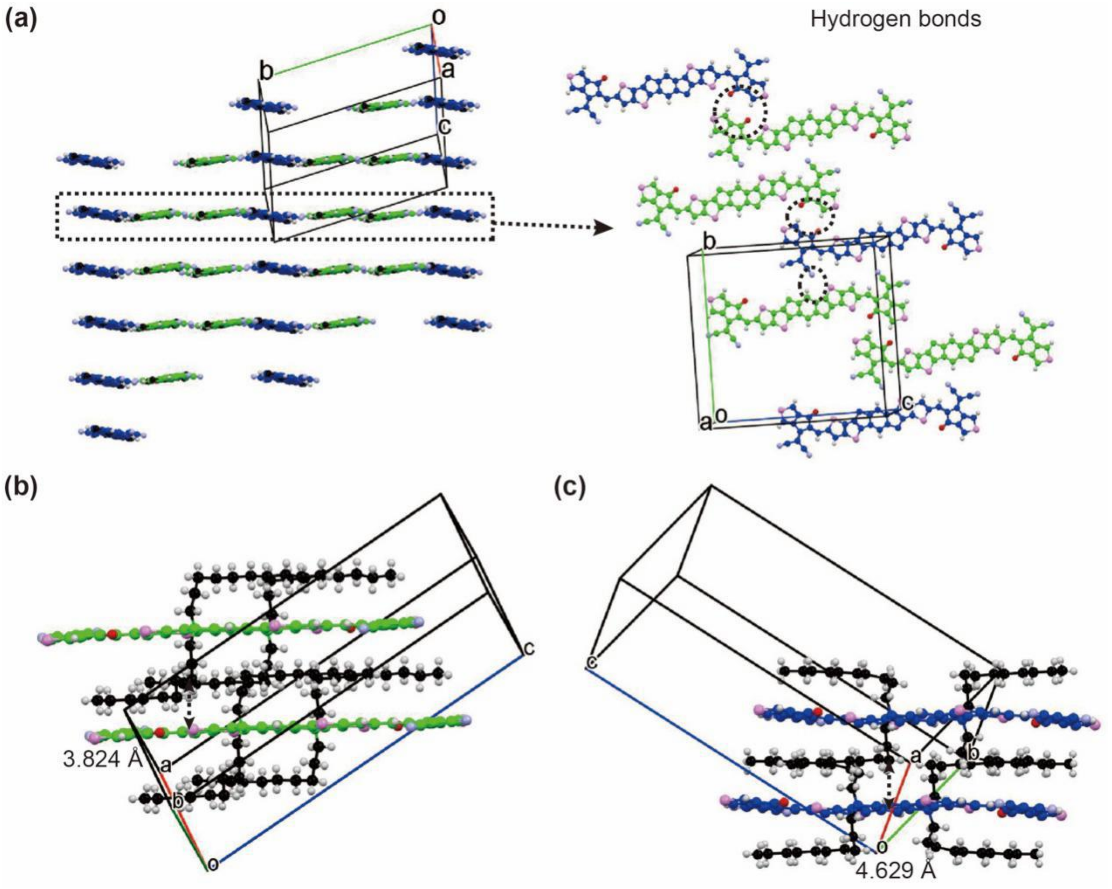

Supplementary Figure 10. a IDTT-C10-TIC packing and hydrogen bonding interactions. Two backbone layers have a large distance of around 7 Å. **b, c** Two pairs of interactions constitute by the blue and green conformers. The distances from side chain to IDTT-C10-TIC backbone are around 3.0-4.0 Å.

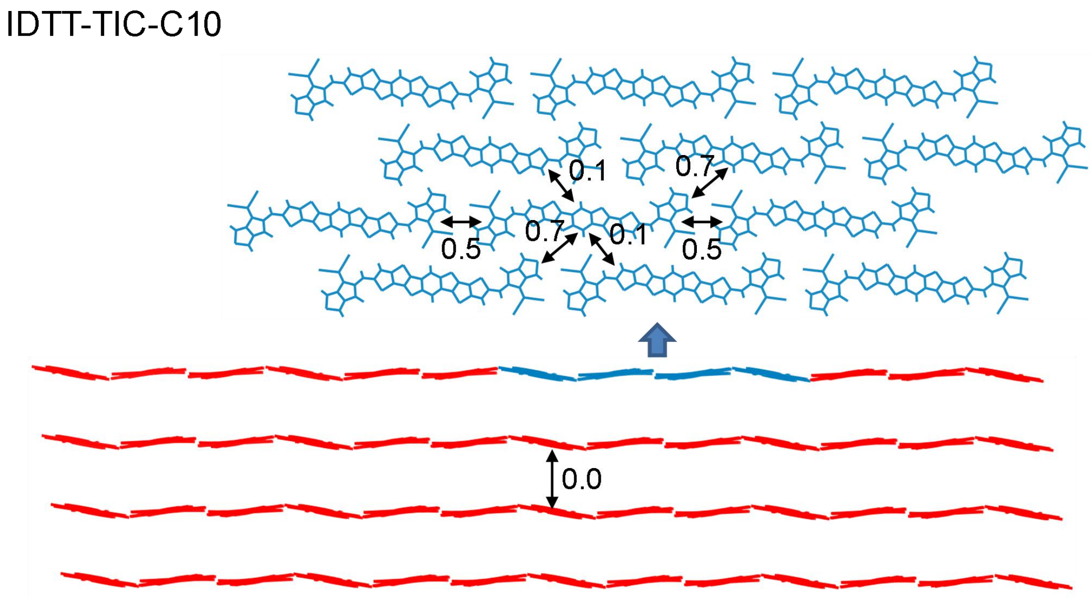

Supplementary Figure 11. Crystal packing and electronic coupling for IDTT-C10-TIC. The transfer integral values between two backbone layers are almost 0 meV. The hydrogen bonds also produce small transfer integral values.

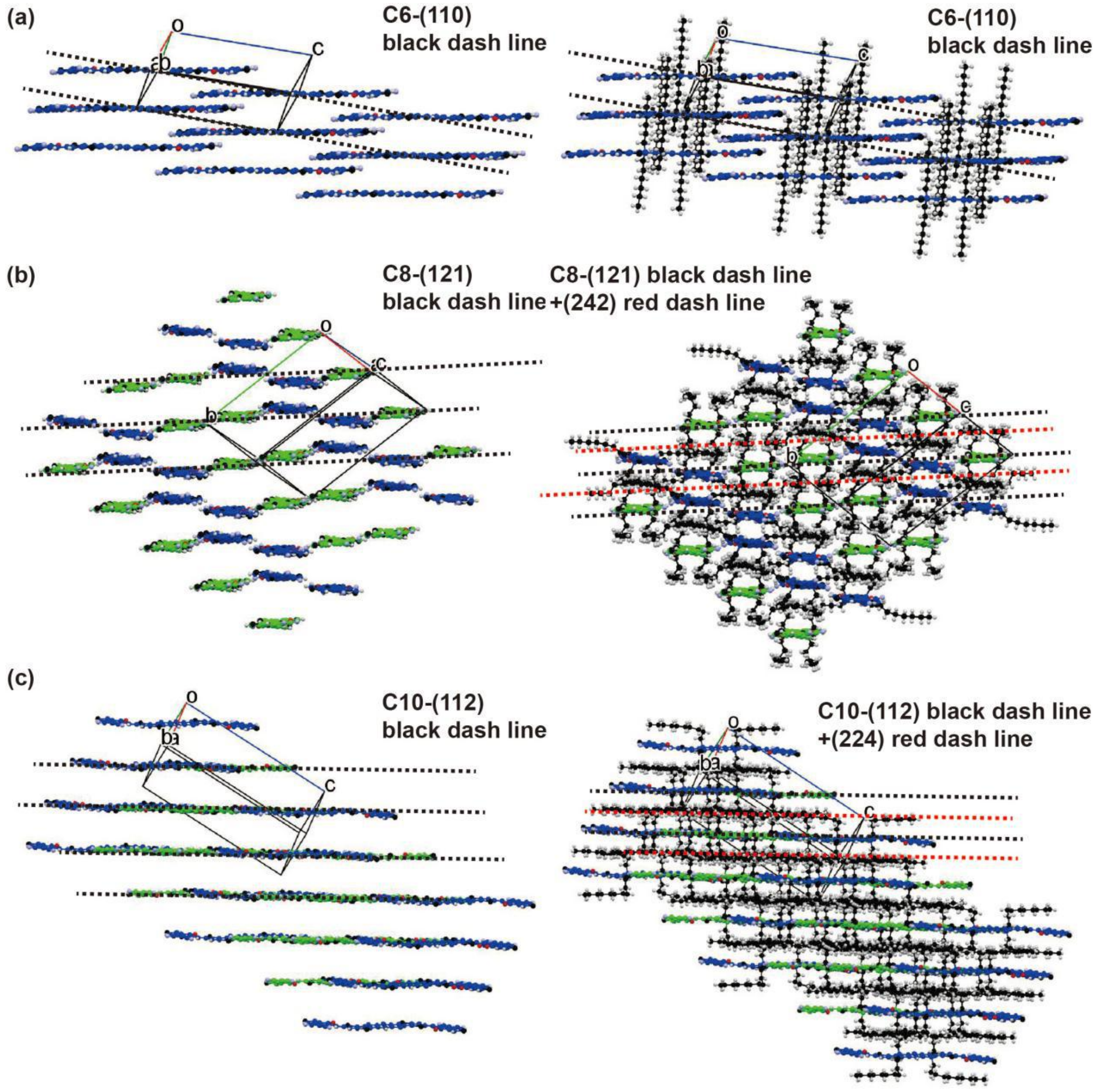

Supplementary Figure 12. Crystal plane of IDTT-CX-TIC without (left), and with (right) side chains. **a** IDTT-C6-TIC (110) crystal plane. **b** IDTT-C8-TIC (121) and (242) crystal planes. **c** IDTT-C10-TIC (112) and (224) crystal planes.

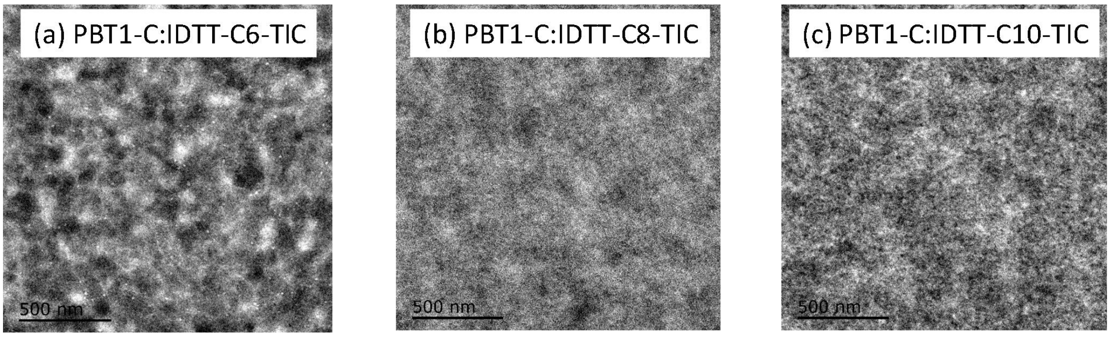

Supplementary Figure 13. TEM images of PBT1-C:IDTT-CX-TIC blends. **a** PBT1-C:IDTT-C6-TIC blends show large-sized aggregations. Through RSoXS analysis, the largest domain size of about 200 nm. **b** IDTT-C8-TIC and **c** IDTT-C10-TIC blends show uniform films with appropriate domain size.

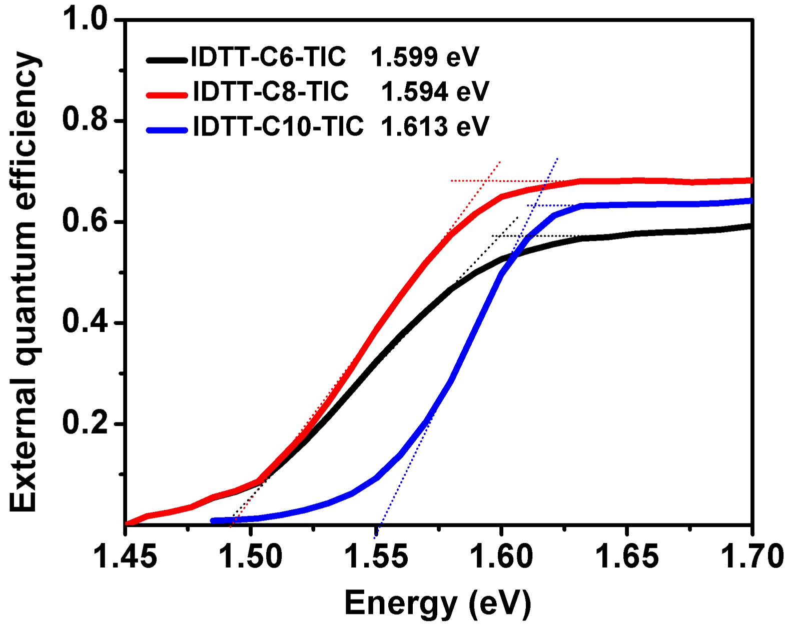

Supplementary Figure 14. Determination of bandgap from the intersection of the absorption edge and the local EQE maximum. The intersection point crossed by the absorption edge tangent and maximum EQE level tangent. Choosing this method can avoid underestimation the bandgap value as a tail state can be observed from three EQE curves.

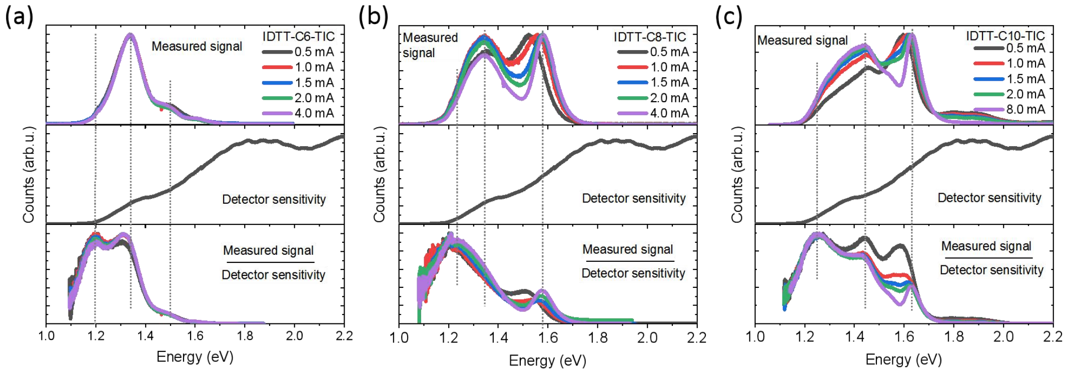

Supplementary Figure 15. Comparison of electroluminescence spectra before and after correction with detector sensitivity. Normalized measured signal (top), detector sensitivity (middle) and normalized corrected spectra by dividing measured signal by detector sensitivity for devices based on **a** IDTT-C6-TIC, **b** IDTT-C8-TIC and **c** IDTT-C10-TIC. It is noted that Si CCD detector is used in EL measurement. A general way to correct spectra is to divide the measured signal by detector sensitivity. While the detector has good sensitivity in the range of acceptor exciton emission, the sensitivity at lower energy part of charge transfer state emission below 1.3 eV is low. After correction of the measured signal by dividing detector sensitivity, this lower energy part of charge transfer states gets much more pronounced. Although this detector sensitivity correction is not suitable for low energy part, it is necessary for the whole spectra in order to get correct peak positions. So we used the corrected spectra for decomposition of emissions from acceptor excitons and charge transfer states with the emission below 1.3 eV as one fitting peak to account for vibrations of the main charge transfer states emission peak (above 1.3 eV).

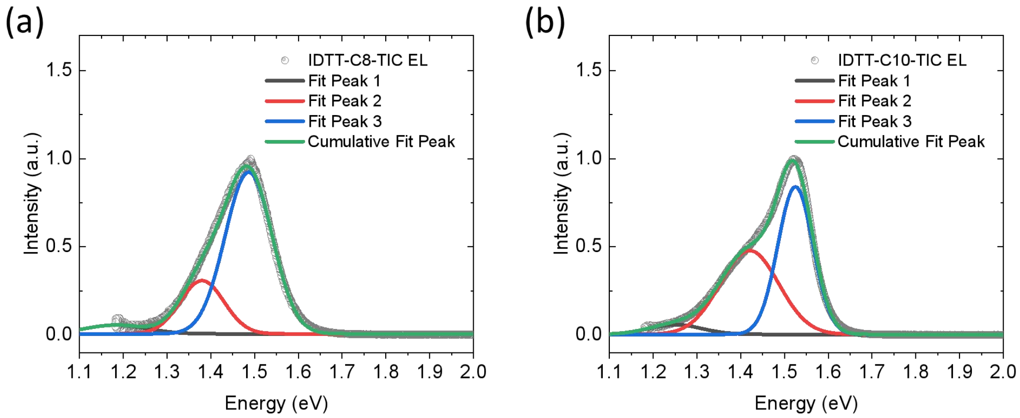

Supplementary Figure 16. Normalized EL and corresponding fits for devices based on **a** IDTT-C8-TIC and **b** IDTT-C10-TIC pristine films.

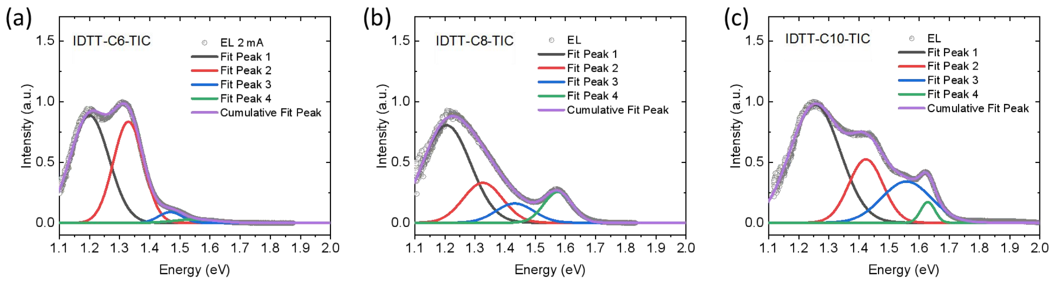

Supplementary Figure 17. a-c EL peak decomposition of IDTT-CX-TIC blends. The EL spectra of blend were fitted using 4 Gaussian peaks according to the equation $y = y_0 + \sum_{i=1}^{4} A_i * e^{\frac{(x-x_{ci})^2}{2{w_i}^2}}$, y_0 is spectra offset, A is peak amplitude, x_c is the peak center energy, w is the width of the peak. The two high energy fitting peaks are assigned to singlet emission with vibrational features, and the two fitting peaks at lower energy are from charge transfer states emission with vibrational features. The peak center energy and peak width of the fitting components (shown in the Supplementary Table S9) are fitted with information from the not corrected spectra where the main CT state emission peak is dominant.

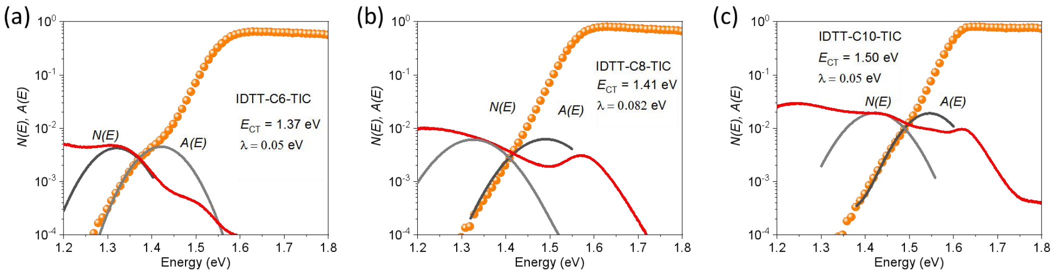

Supplementary Figure 18. Fitting of charge transfer state emission (*N(E)*) and absorption (*A(E)*) according to Marcus theory[1]: $N(E) \propto E exp[-\frac{(E_{CT}-\lambda-E)^2}{4\lambda k_B T}]$, $A(E) \propto \frac{1}{E} exp[-\frac{(E_{CT}+\lambda-E)^2}{4\lambda k_B T}]$. Here E is energy, E_{CT} is charge transfer state energy and λ is reorganization energy. The fittings for devices based on **a** IDTT-C6-TIC, **b** IDTT-C8-TIC and **c** IDTT-C10-TIC as acceptors. Red line is reduced emission spectra, orange dots line are the reduced absorption spectra, dark and light gray line are the corresponding fits of the high energy part of charge transfer state emission and lower energy part of charge transfer state absorption. Charge transfer state energy (E_{CT}) and reorganization energy (λ) are indicated in the graph inset.

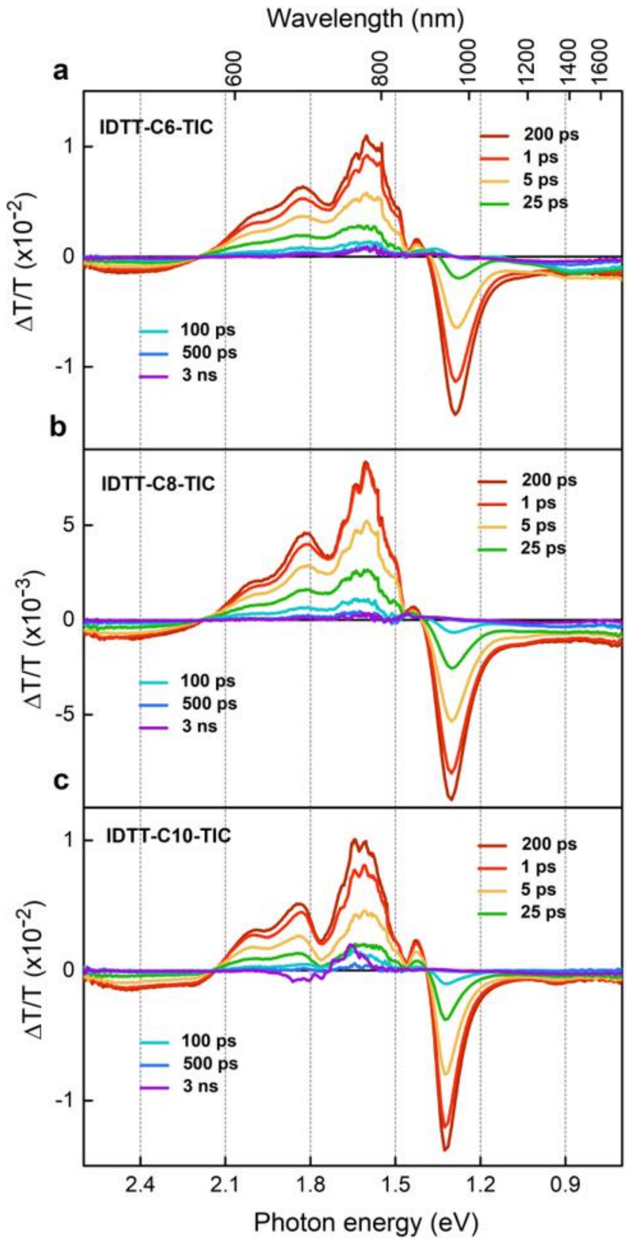

Supplementary Figure 19. Transient absorption spectra of IDTT-CX-TIC pristine films at different pump-probe delay times after the excitation at 710 nm. **a** IDTT-C6-TIC (5.1 μJ/cm^2), **b** IDTT-C8-TIC (4.7 μJ/cm^2) and **c** IDTT-C10-TIC (5.8 μJ/cm^2).

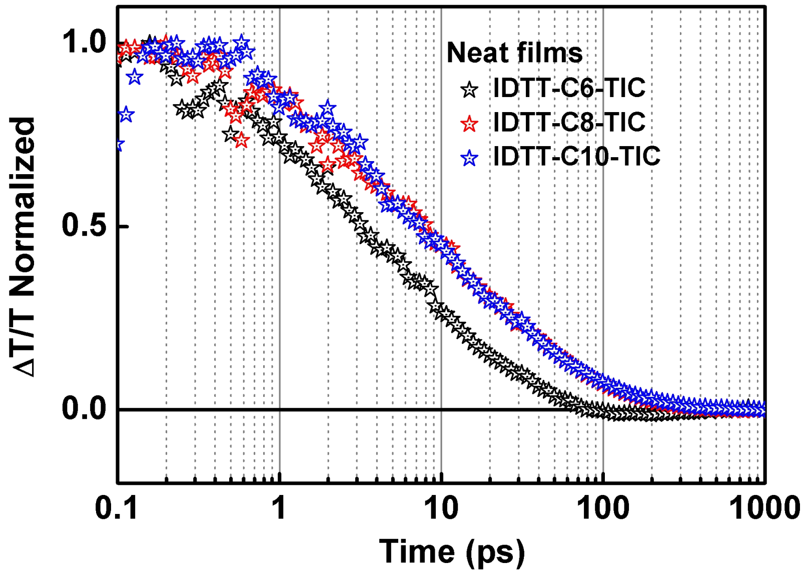

Supplementary Figure 20. Transient absorption kinetics of excitons in IDTT-CX-TIC pristine films.

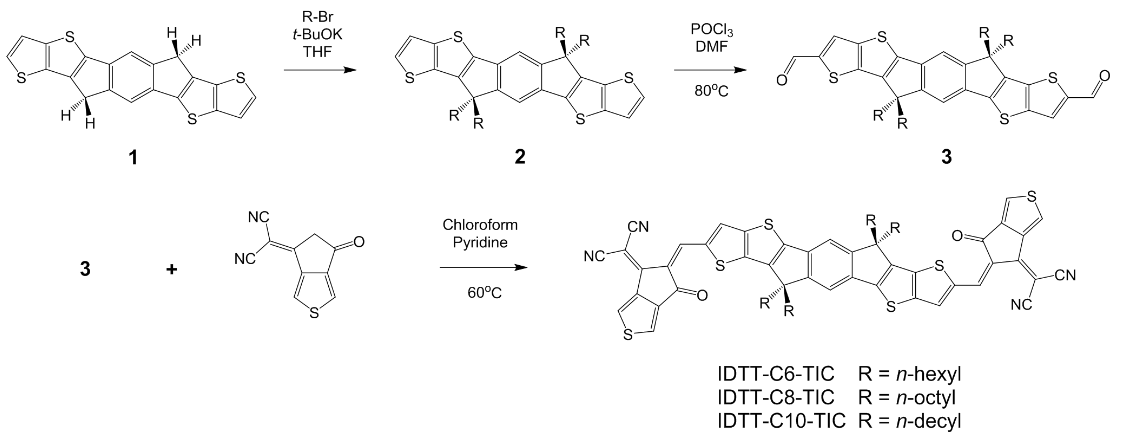

Supplementary Figure 21. Synthetic routes of IDTT-CX-TIC.

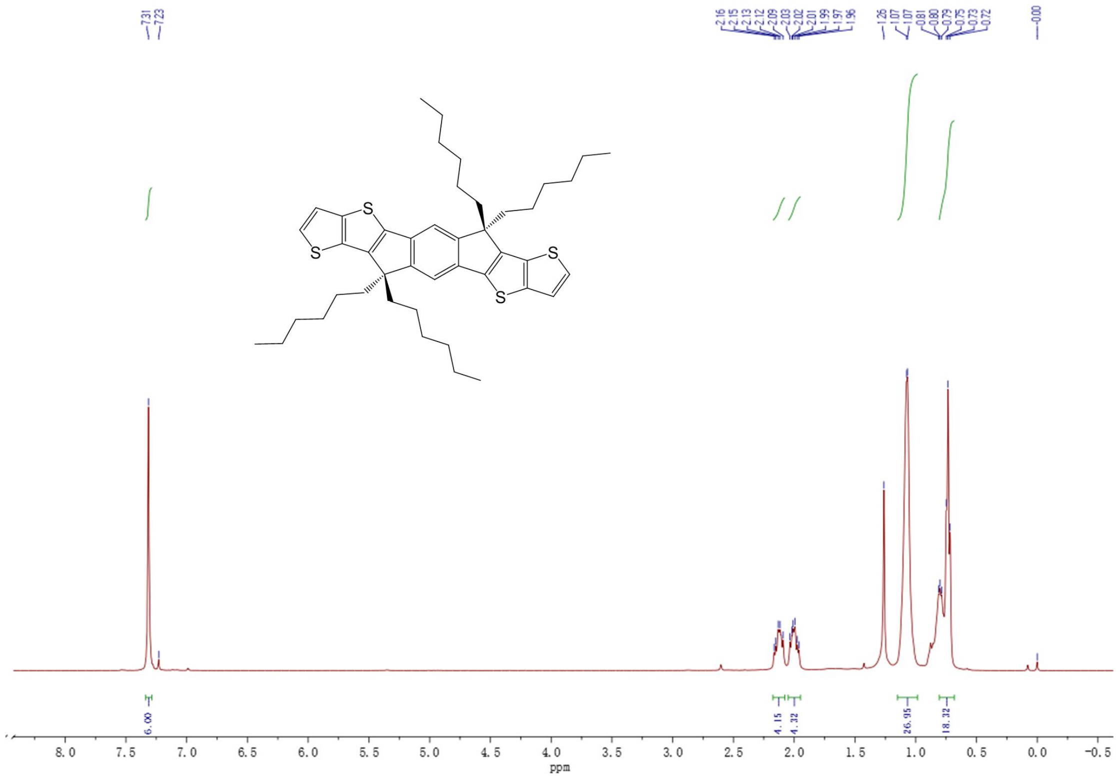

Supplementary Figure 22. ^{1}H NMR spectrum of hexyl 2.

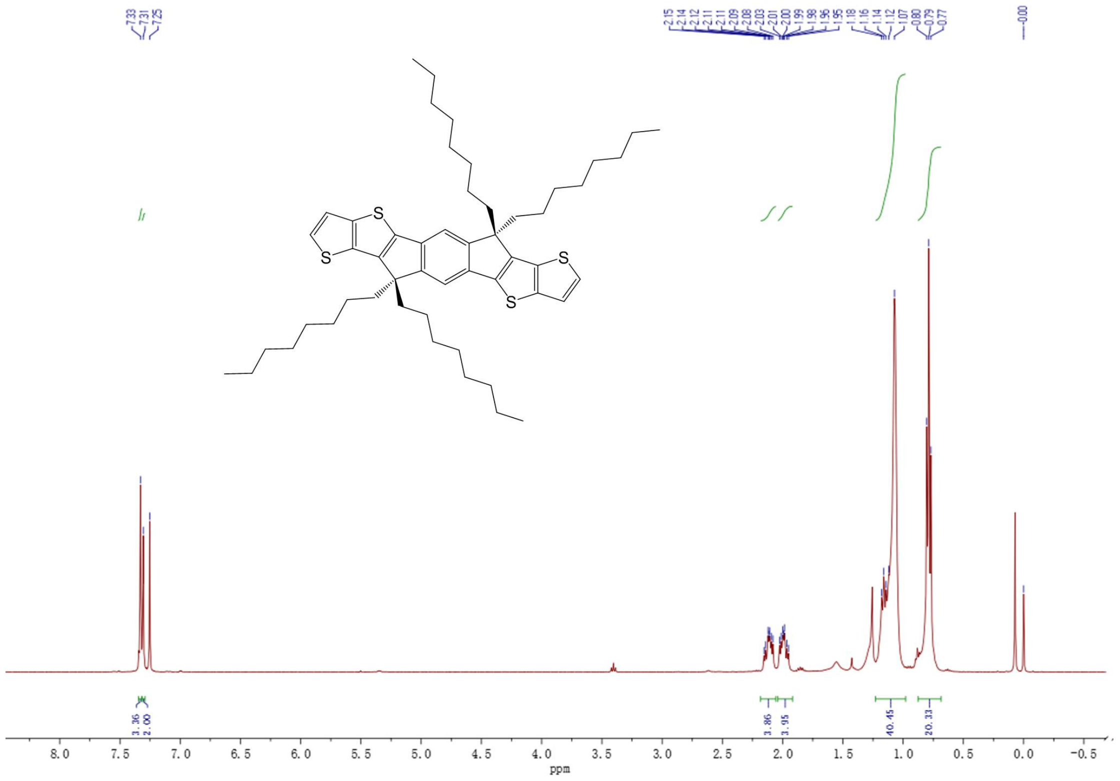

Supplementary Figure 23. ^{1}H NMR spectrum of octyl 2.

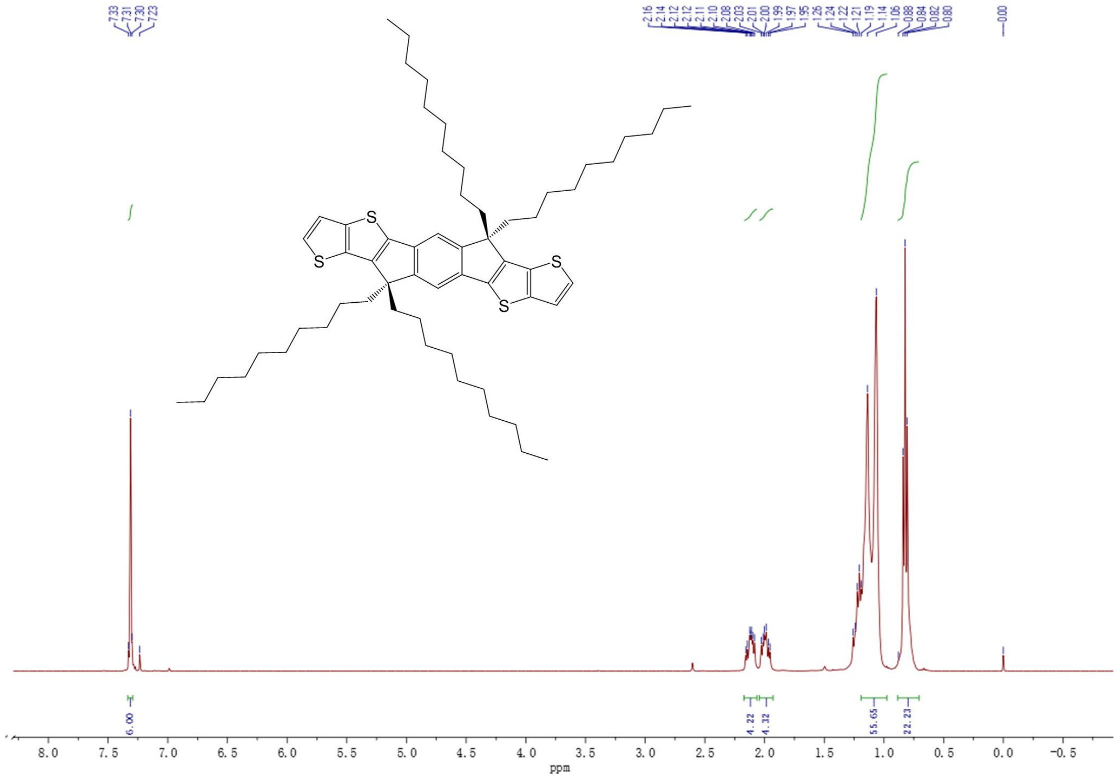

Supplementary Figure 24. ^{1}H NMR spectrum of decyl 2.

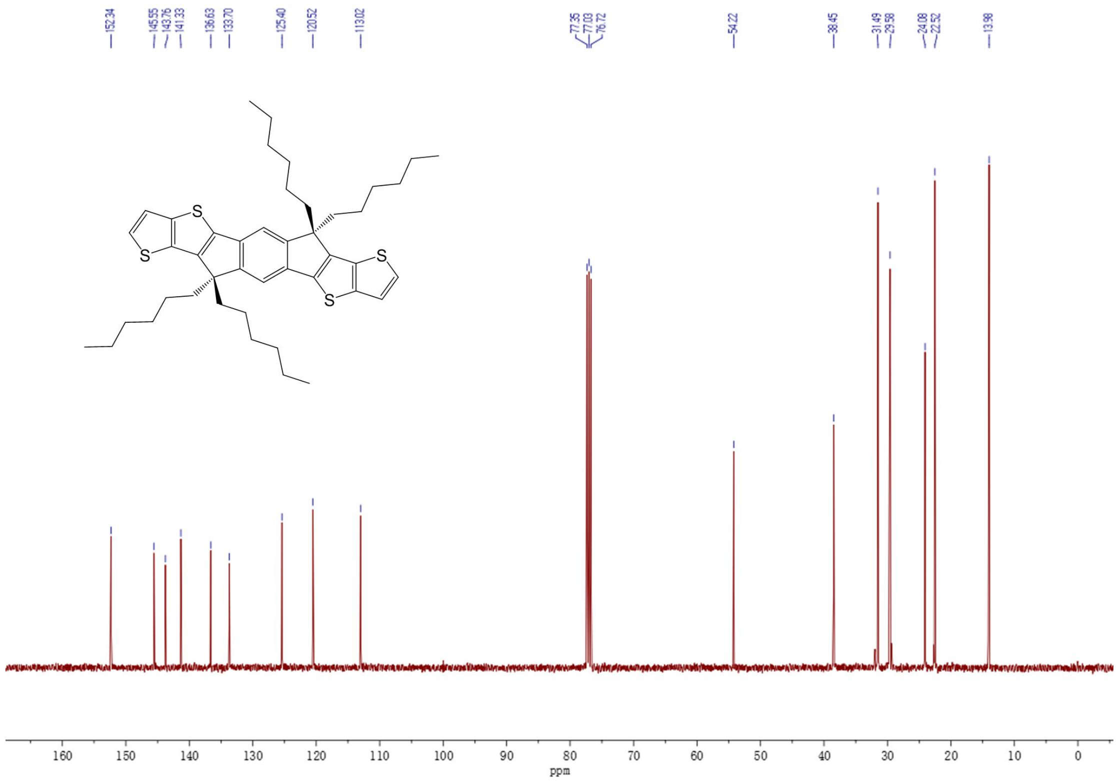

Supplementary Figure 25. ^{13}C NMR spectrum of hexyl 2.

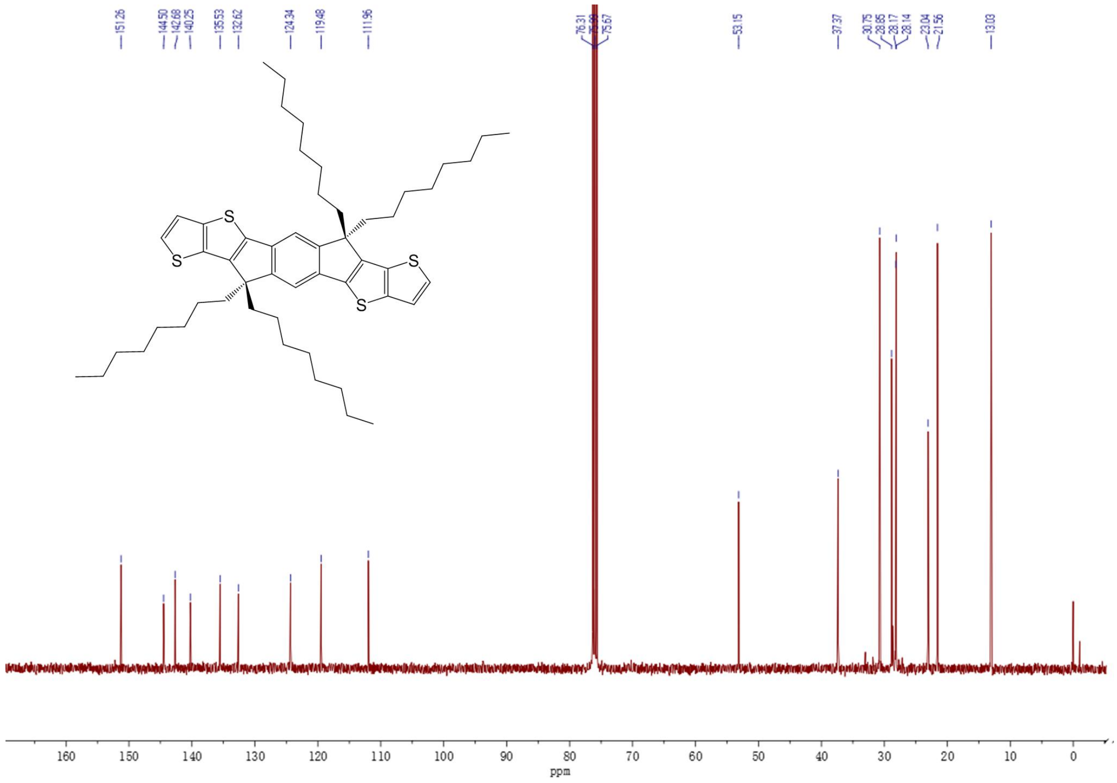

Supplementary Figure 26. ^{13}C NMR spectrum of octyl 2.

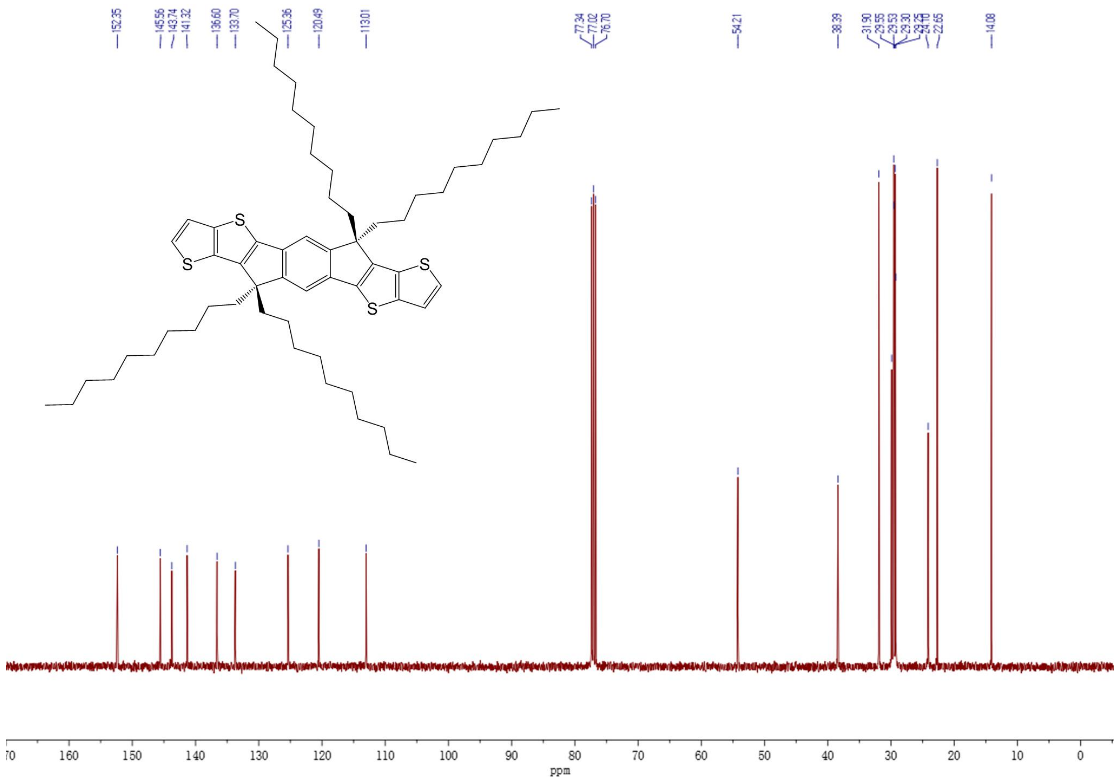

Supplementary Figure 27. ^{13}C NMR spectrum of decyl 2.

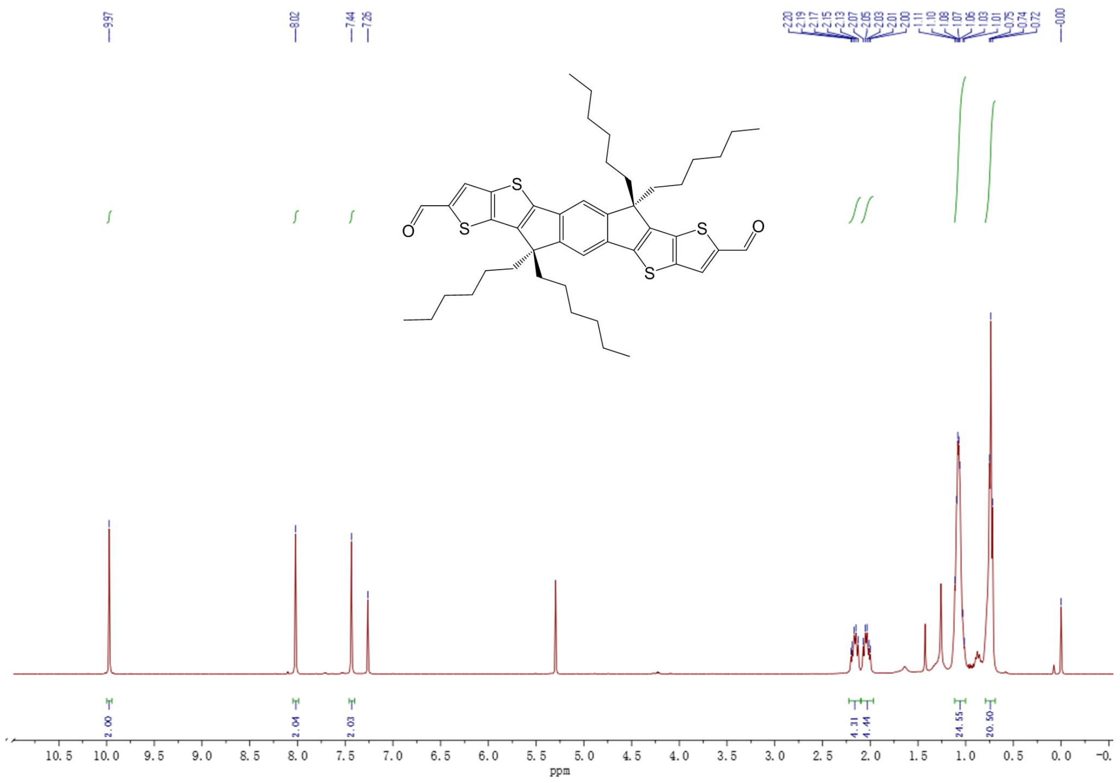

Supplementary Figure 28. ^{1}H NMR spectrum of hexyl 3.

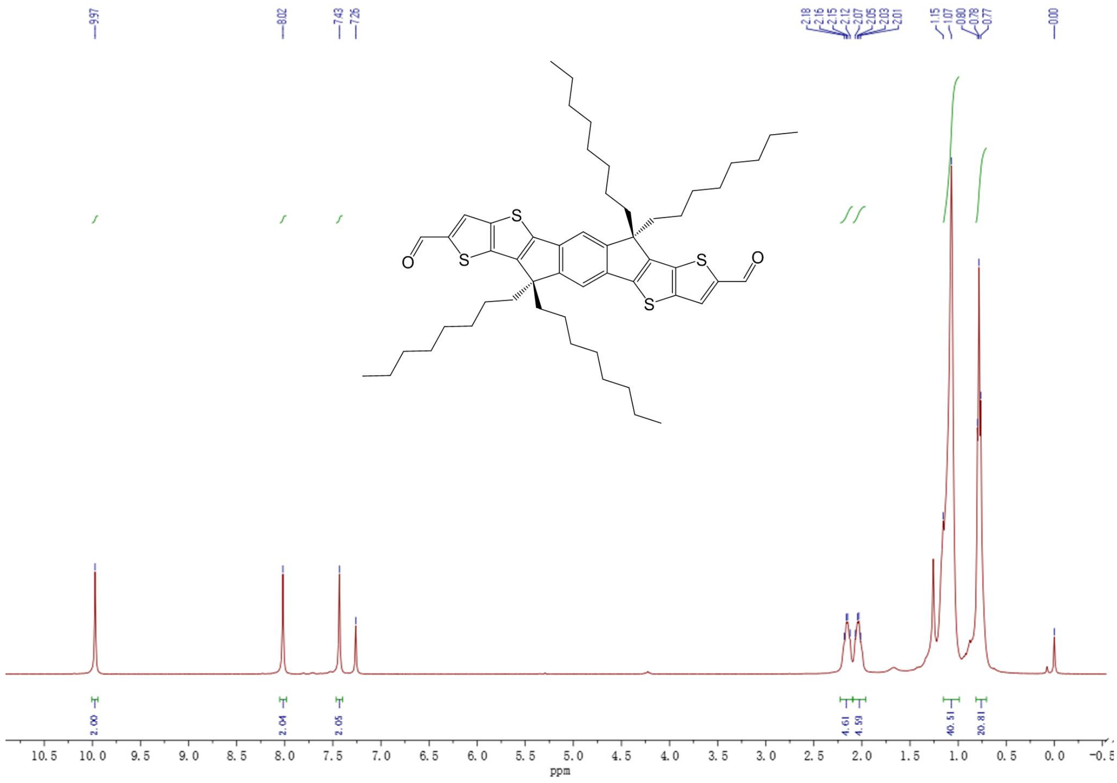

Supplementary Figure 29. ^{1}H NMR spectrum of octyl 3.

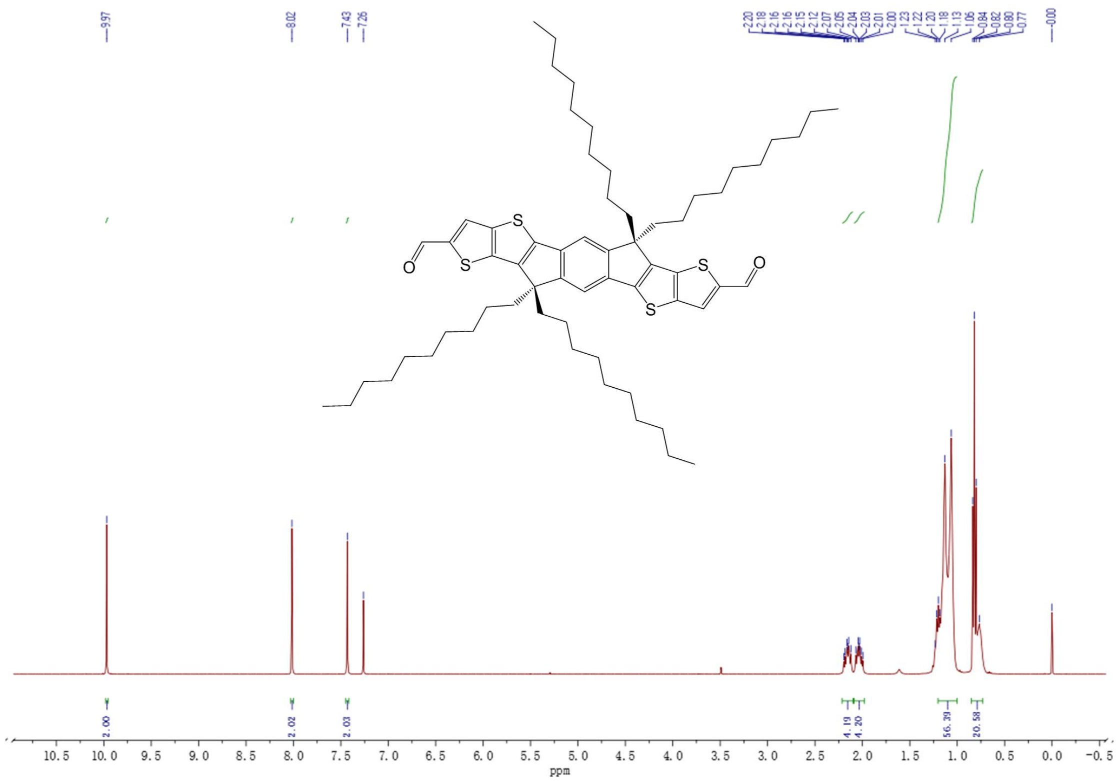

Supplementary Figure 30. ^{1}H NMR spectrum of decyl 3.

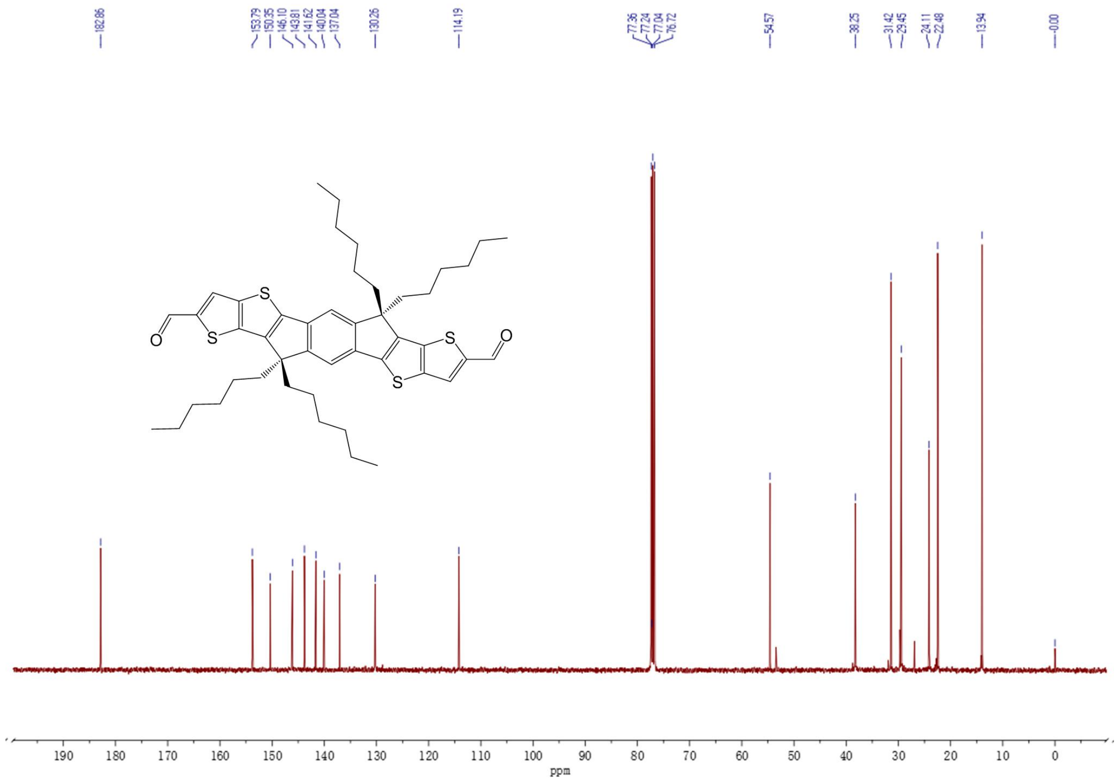

Supplementary Figure 31. ^{13}C NMR spectrum of hexyl 3.

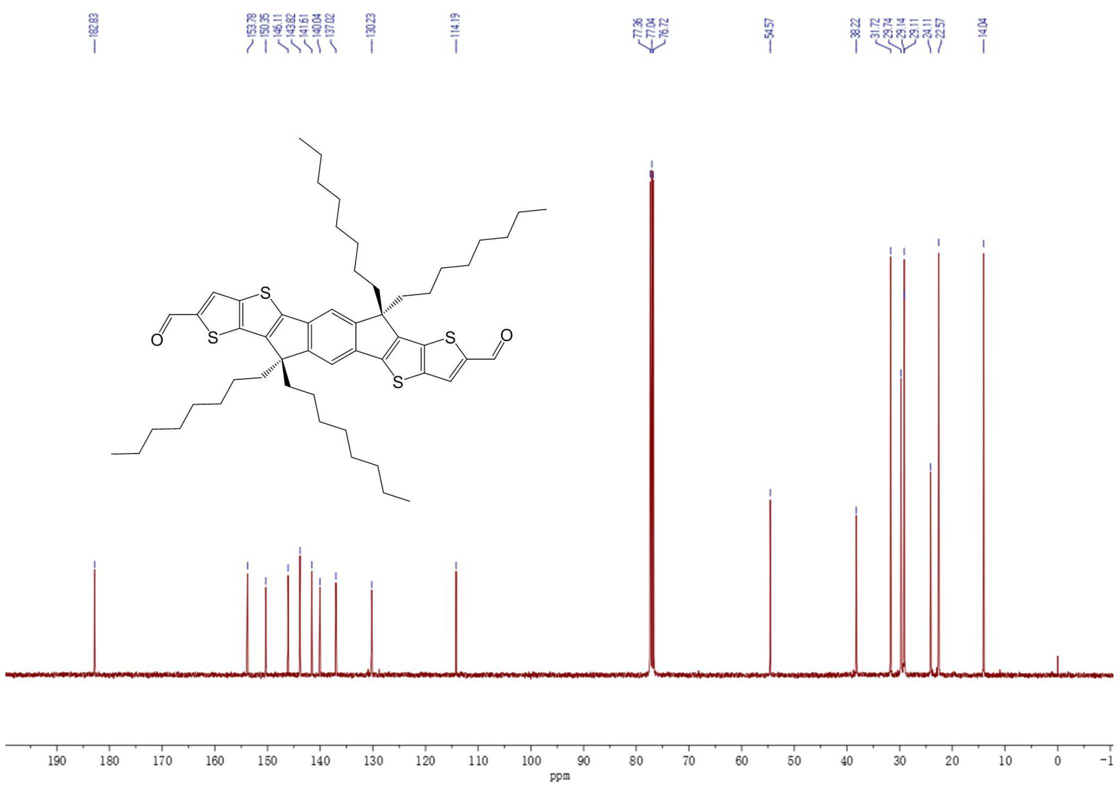

Supplementary Figure 32. ^{13}C NMR spectrum of octyl 3.

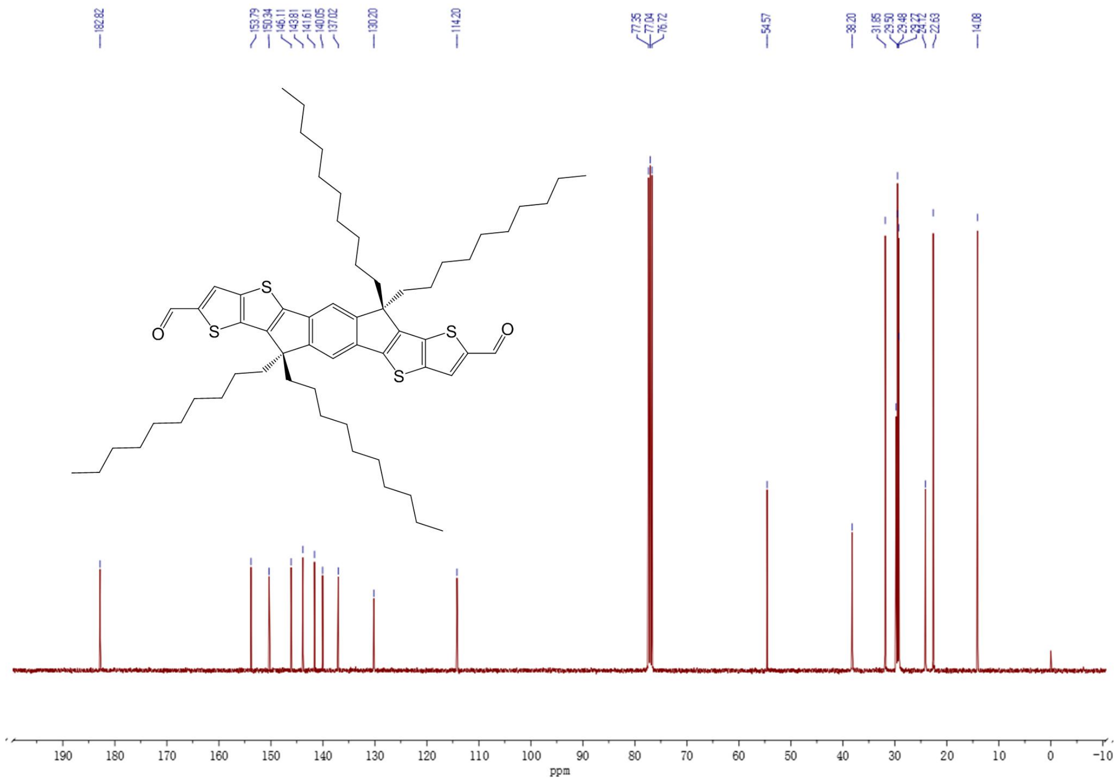

Supplementary Figure 33. ^{13}C NMR spectrum of decyl 3.

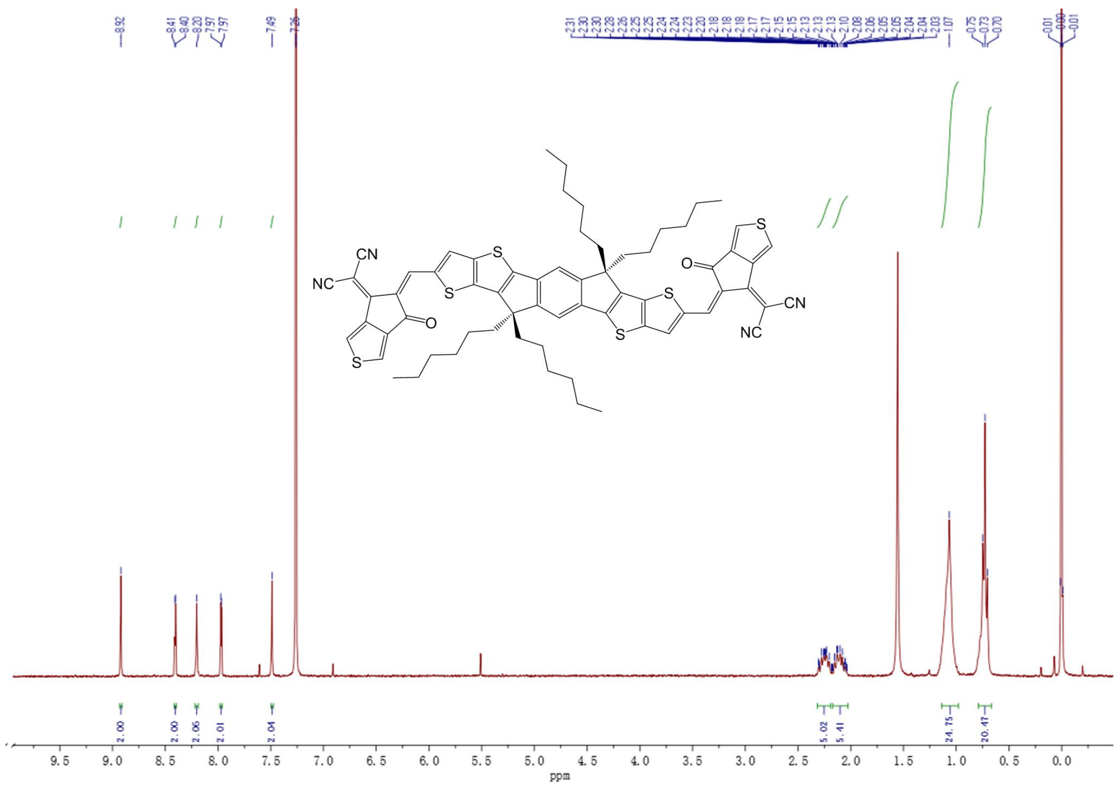

Supplementary Figure 34. ^{1}H NMR spectrum of IDTT-C6-TIC.

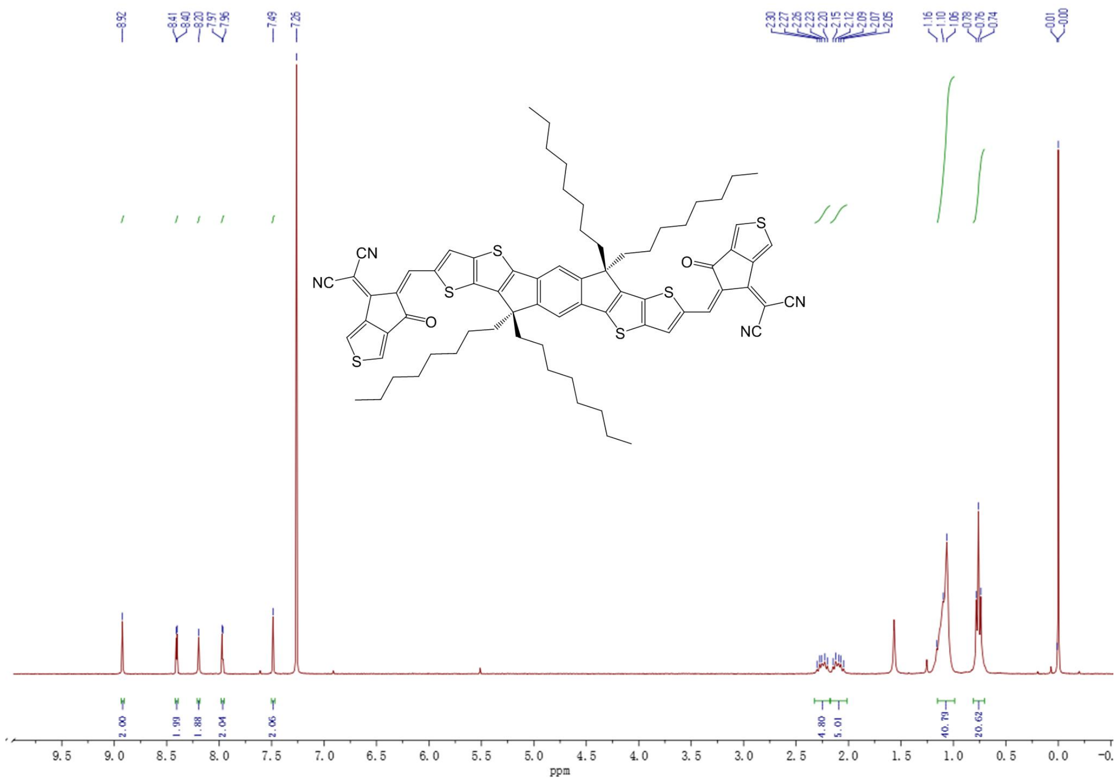

Supplementary Figure 35. ^{1}H NMR spectrum of IDTT-C8-TIC.

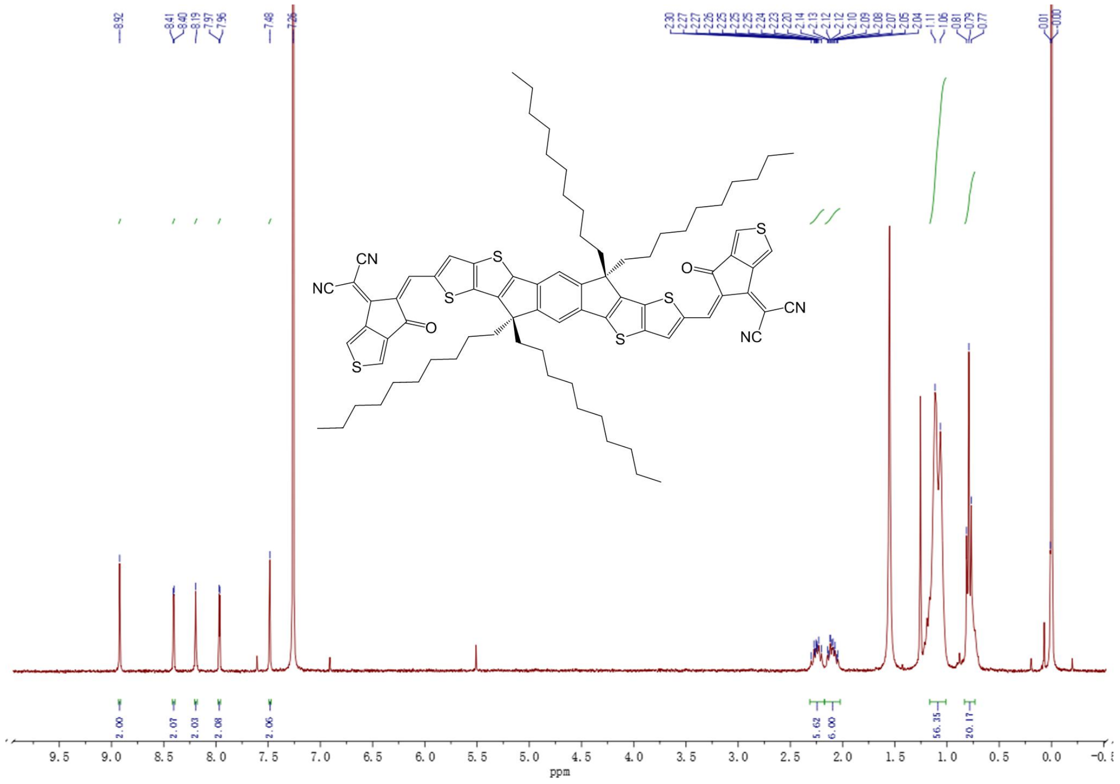

Supplementary Figure 36. ^{1}H NMR spectrum of IDTT-C10-TIC.

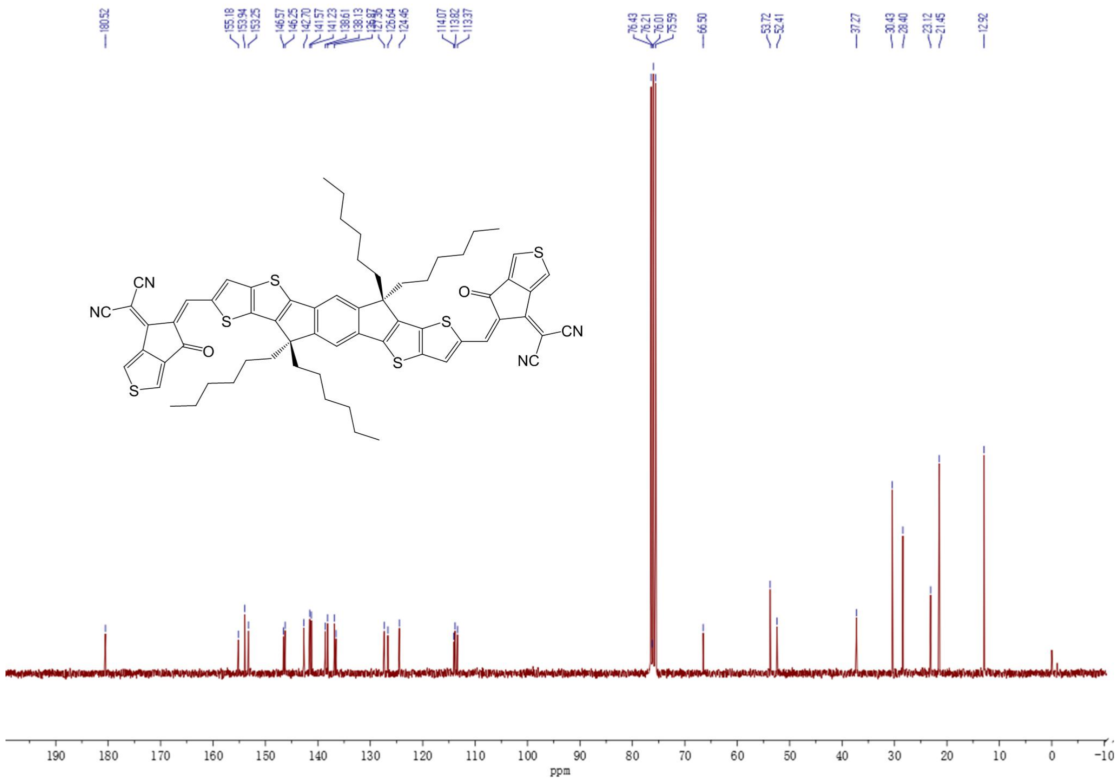

Supplementary Figure 37. ^{13}C NMR spectrum of IDTT-C6-TIC.

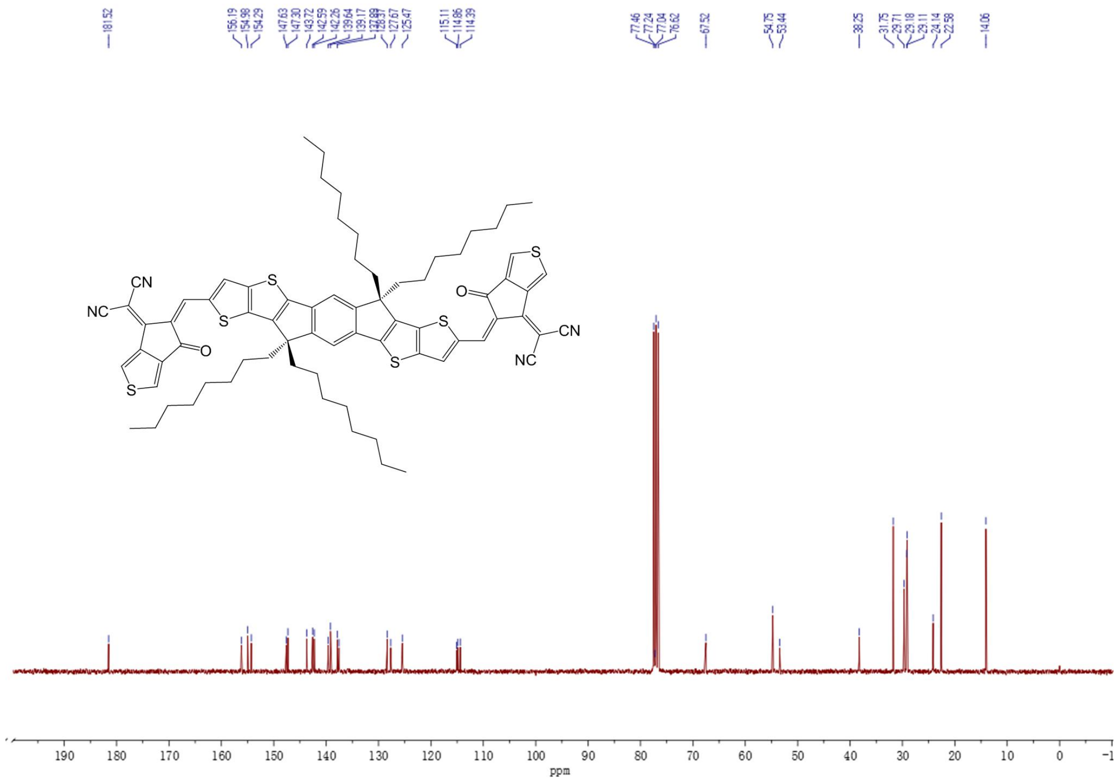

Supplementary Figure 38. ^{13}C NMR spectrum of IDTT-C8-TIC.

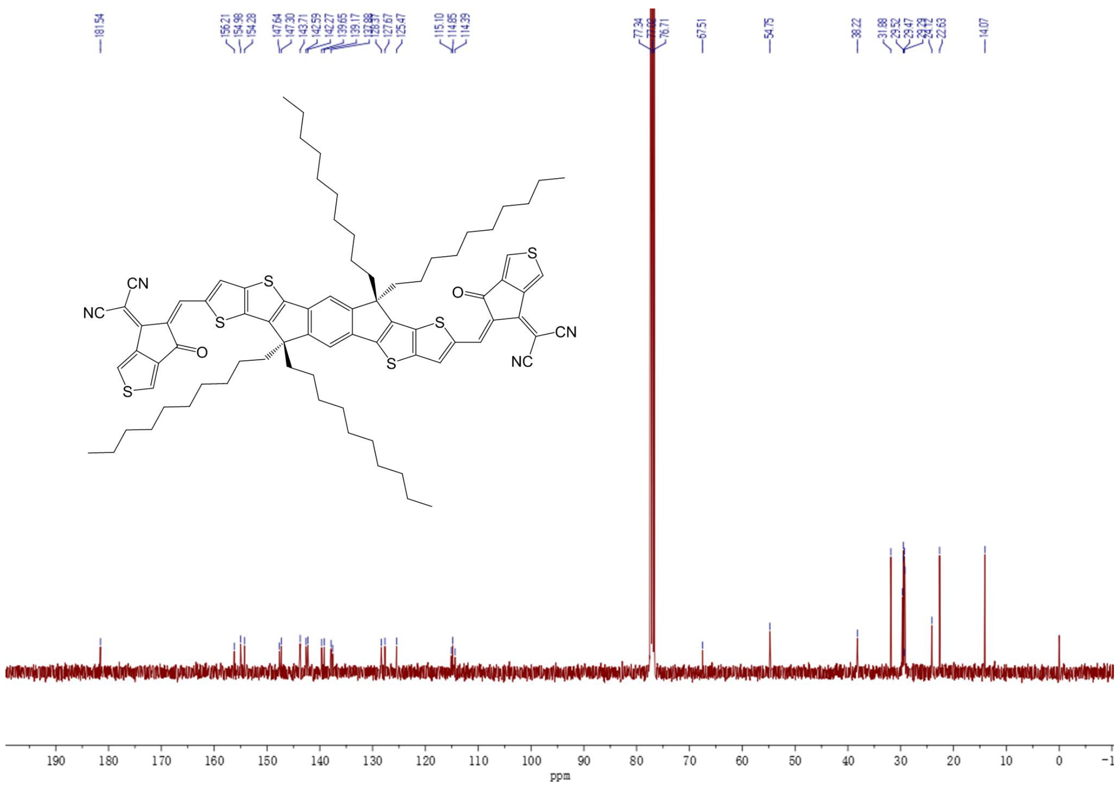

Supplementary Figure 39. ^{13}C NMR spectrum of IDTT-C10-TIC.

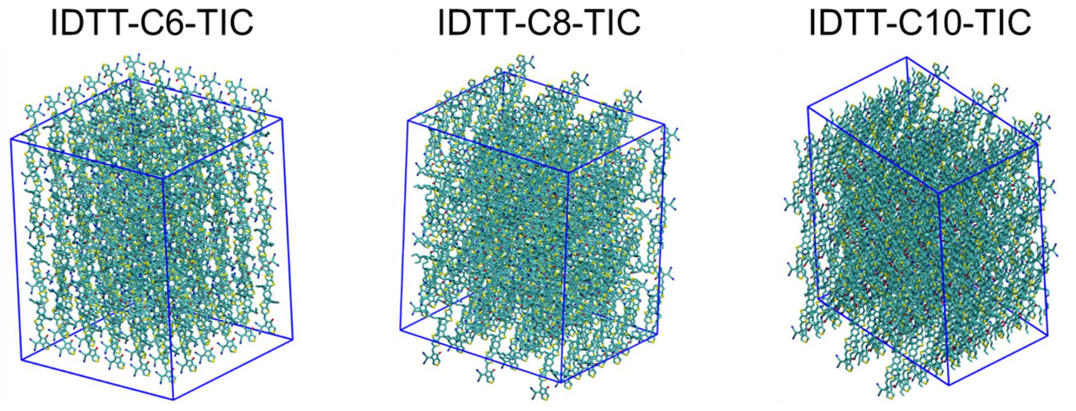

Supplementary Figure 40. Supercell for IDTT-CX-TIC. The inherent hydrogen atoms are omitted for clarity.

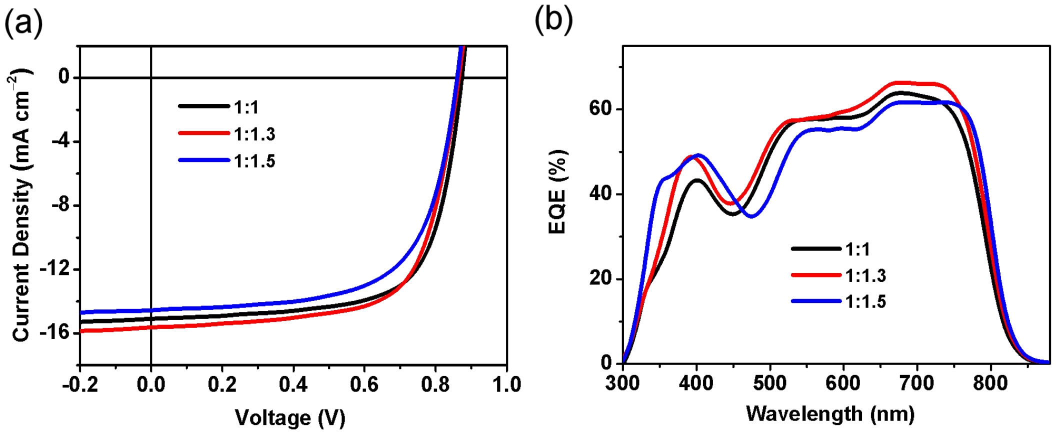

Supplementary Figure 41. **a** *J–V* characteristics and **b** the corresponding EQE spectra of PBT1-C:IDTT-C6-TIC solar cells with different blend ratios.

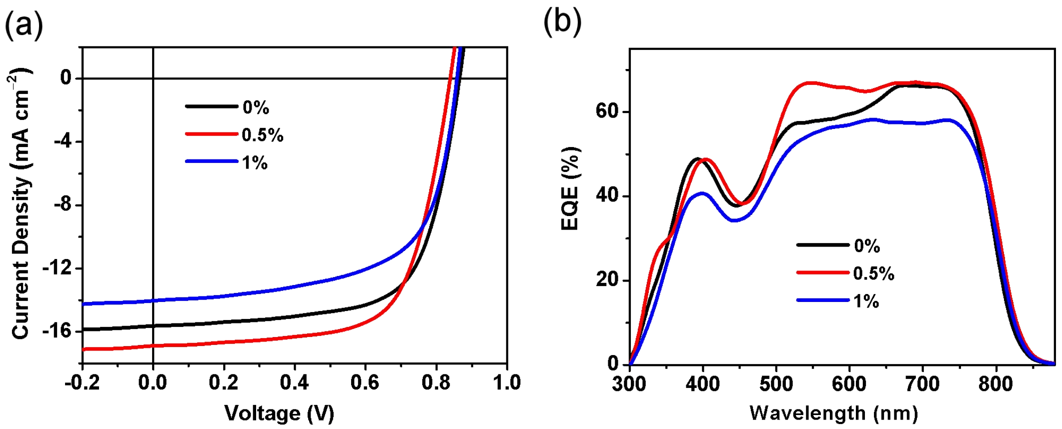

Supplementary Figure 42. **a** *J–V* characteristics and **b** the corresponding EQE spectra of PBT1-C:IDTT-C6-TIC solar cells with different DIO contents.

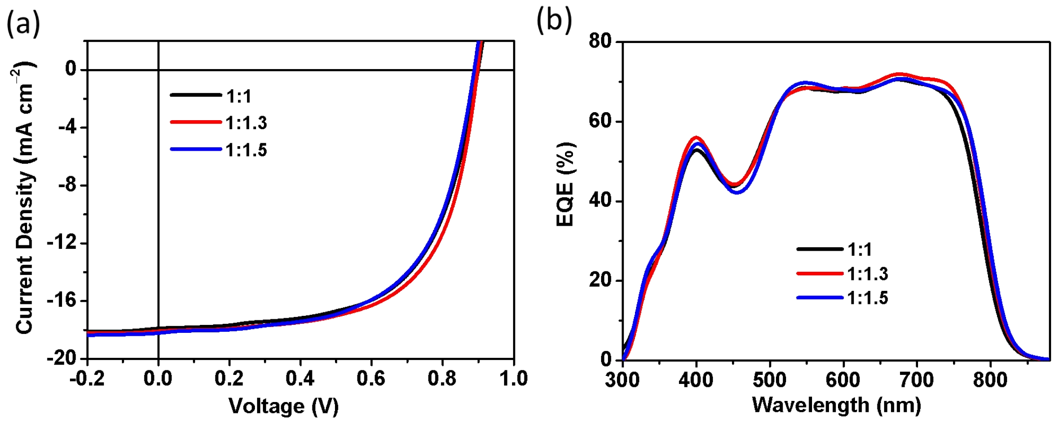

Supplementary Figure 43. **a** *J–V* characteristics and **b** the corresponding EQE spectra of PBT1-C:IDTT-C8-TIC solar cells with different blend ratios.

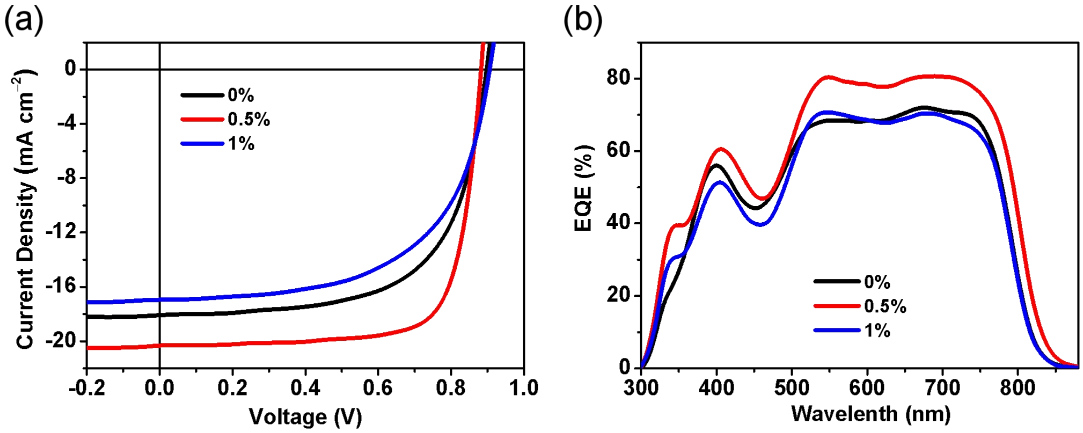

Supplementary Figure 44. **a** *J–V* characteristics and **b** the corresponding EQE spectra of PBT1-C:IDTT-C8-TIC solar cells with different DIO contents.

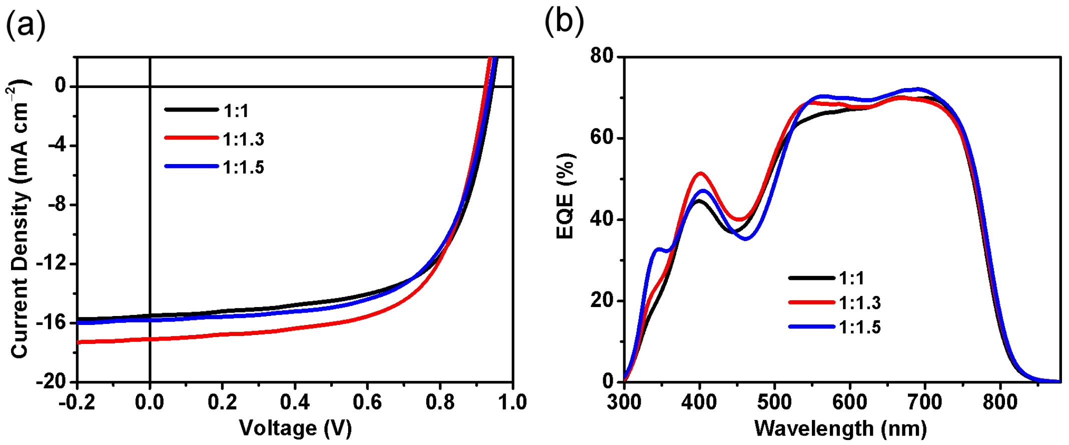

Supplementary Figure 45. a *J–V* characteristics and **b** the corresponding EQE spectra of PBT1-C:IDTT-C10-TIC solar cells with different blend ratios.

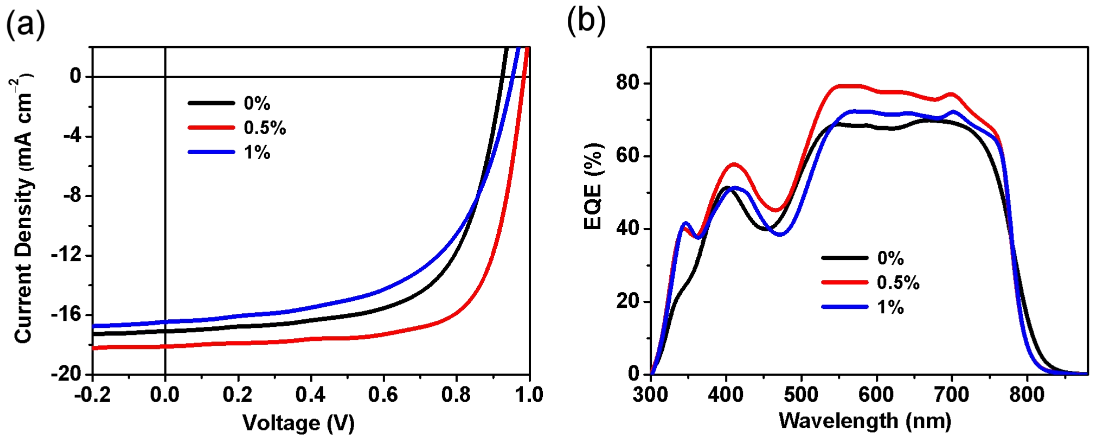

Supplementary Figure 46. **a** *J–V* characteristics and **b** the corresponding EQE spectra of PBT1-C:IDTT-C10-TIC solar cells with different DIO contents.

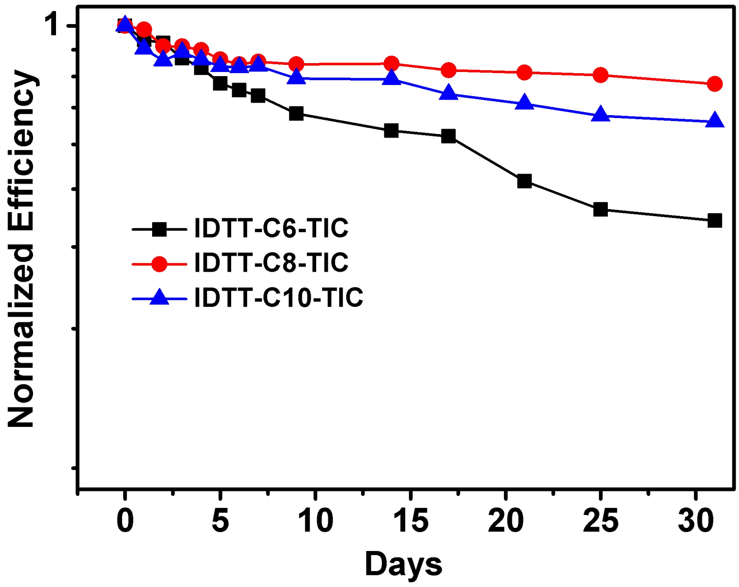

Supplementary Figure 47. Degradation of normalized PCEs of OSCs based on PBT1-C:IDTT-CX-TIC blends. The devices were unencapsulated and stored in air for 30 days.

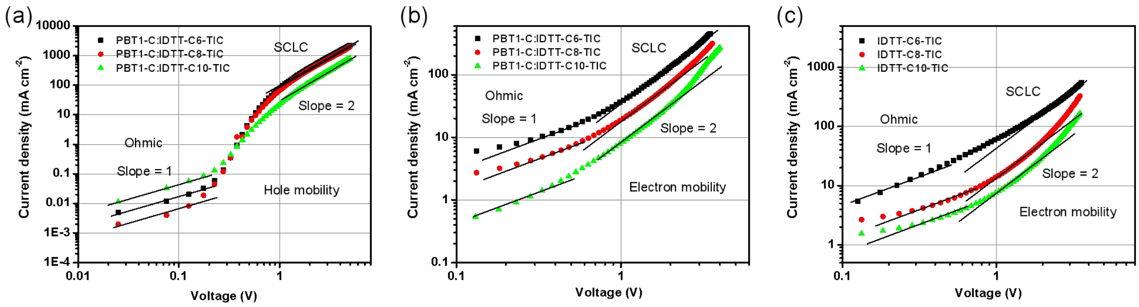

Supplementary Figure 48. Characteristic curves of **a** hole-only and **b** electron-only SCLC devices based on PBT1-C:IDTT-CX-TIC blends, and **c** hole-only SCLC devices based on PBT1-C:IDTT-CX-TIC pristine films.

Supplementary Tables

Supplementary Table 1. Electrochemical data of IDTT-CX-TIC.

Materials	HOMO (eV)	LUMO (eV)
IDTT-C6-TIC	-5.55	-3.99
IDTT-C8-TIC	-5.64	-3.97
IDTT-C10-TIC	-5.71	-3.91

Supplementary Table 2. Electron mobilities for crystals and thin films.

μ_e ($cm^2\ V^{-1}\ s^{-1}$)	Crystal (simulated)	Thin film (measured)
IDTT-C6-TIC	2.4×10^{-3}	1.2×10^{-3}
IDTT-C8-TIC	2.9×10^{-4}	2.2×10^{-4}
IDTT-C10-TIC	6.5×10^{-5}	9.8×10^{-5}

Supplementary Table 3. GIWAXS data of IDTT-C6-TIC neat and blend films.

Neat film	Location (Å^{-1}) and crystal plane	Area	CCL (nm)	Blend film	Location (Å^{-1}) and crystal plane	Area	CCL (nm)
In plane	0.396 (001)	11.67	14.272	In plane	0.281	40.84	9.547
	0.600 (100/010)	3.711	16.771		0.371 (001)	18.711	10.351
	0.725 (1-10)	3.357	10.95		0.725 (1-10)	8.897	3.980
	1.644	3.594	5.981		1.893	10.553	3.229
	1.855	3.978	4.095				
Out of plane	0.388 (001)	9.810	10.953	Out of plane	0.299	32.28	9.028
	0.597 (100/010)	6.267	15.400		0.714 (1-10)	6.400	4.186
	0.730 (1-10)	8.019	10.624		1.744	67.818	1.293
	1.854	6.641	3.286		1.913	9.242	4.789

Supplementary Table 4. GIWAXS data of IDTT-C8-TIC neat and blend films.

Neat film	Location (Å^{-1}) and crystal plane	Area	CCL (nm)	Blend film	Location (Å^{-1}) and crystal plane	Area	CCL (nm)
In plane				In plane	0.281	29.078	9.694
	0.380 (01-1)	5.001	12.758		0.374 (01-1)	18.632	8.985
Out of plane				Out of plane	0.300	29.816	10.202
	0.623 (021)	3.364	14.381		0.354	2.492	8.602
	0.814 (121)	0.569	15.618		0.858	1.6801	11.198
	1.605 (242)	11.043	6.090		1.747	21.395	2.959
	1.889	73.904	1.771		1.924	27.812	3.767

Supplementary Table 5. GIWAXS data of IDTT-C10-TIC neat and blend films.

Neat film	Location ($Å^{-1}$) and crystal plane	Area	CCL (nm)	Blend film	Location ($Å^{-1}$) and crystal plane	Area	CCL (nm)
In plane	0.799	3.365	11.060	In plane	0.281	40.414	10.428
	0.857	2.720	10.313		0.801	4.393	10.389
Out of plane				Out of plane	0.306	91.367	7.349
	0.854 (112)	23.268	6.785		0.854 (112)	46.411	4.595
	1.697 (224)	42.569	4.095		1.703	131.5	2.770

Supplementary Table 6. Crystal data and structure refinement for IDTT-CX-TIC.

Identification code	IDTT-C6-TIC	IDTT-C8-TIC	IDTT-C10-TIC
Empirical formula	$C_{66}H_{62}N_4O_2S_6$	$C_{74}H_{78}N_4O_2S_6$	$C_{82}H_{94}N_4O_2S_6$
CCDC number	1974016	1974017	1974018
Formula weight	1135.55	1247.76	1359.97
Temperature/K	106(9)	100.0(10)	100(1)
Crystal system	triclinic	triclinic	triclinic
Space group	P-1	P-1	P-1
a/Å	9.9976(3)	12.0882(2)	8.03983(12)
b/Å	10.1415(3)	22.6796(5)	24.8028(5)
c/Å	20.1032(4)	25.5580(3)	28.2613(4)
α/°	99.401(2)	96.0410(16)	84.4057(13)
β/°	98.738(2)	101.5331(14)	81.9589(12)
γ/°	107.479(3)	101.9925(18)	82.3081(15)
Volume/Å^3	1873.78(10)	6636.3(2)	5512.13(16)
Z	1	4	3
ρ_{calc} g/cm^3	1.006	1.249	1.229
μ/mm^{-1}	1.98	2.28	2.098
F(000)	598	2648	2178
Crystal size/mm^3	0.08 × 0.07 × 0.02	0.08 × 0.05 × 0.02	0.1 × 0.05 × 0.01
Radiation	CuKα (λ = 1.54184)	CuKα (λ = 1.54184)	CuKα (λ = 1.54184)
2Θ range for data collection/°	9.376 to 147.164	4.958 to 134.154	4.606 to 134.146
Index ranges	-11 ≤ h ≤ 12, -12 ≤ k ≤ 6, -24 ≤ l ≤ 24	-13 ≤ h ≤ 14, -26 ≤ k ≤ 27, -30 ≤ l ≤ 30	-9 ≤ h ≤ 9, -21 ≤ k ≤ 29, -28 ≤ l ≤ 33
Reflections collected	17832	60469	53386

Independent reflections	7092 [R_{int} = 0.0375, R_{sigma} = 0.0469]	23057 [R_{int} = 0.0423, R_{sigma} = 0.0498]	19284 [R_{int} = 0.0446, R_{sigma} = 0.0541]
Data/restraints/parameters	7092/0/354	23057/0/1557	19284/23/1289
Goodness-of-fit on F^2	1.062	1.041	1.064
Final R indexes [I>=2σ (I)]	R_1 = 0.0632, wR_2 = 0.1788	R_1 = 0.0879, wR_2 = 0.2310	R_1 = 0.0691, wR_2 = 0.1902
Final R indexes [all data]	R_1 = 0.0676, wR_2 = 0.1840	R_1 = 0.1064, wR_2 = 0.2442	R_1 = 0.0870, wR_2 = 0.2041

Supplementary Table 7. Summary of device parameters of PBT1-C:IDTT-CX-TIC solar cells with different weight ratios under the illumination of AM1.5G, 100 mW cm^{-2}.

Materials	Weight ratio	V_{OC} (V)	J_{SC} (mA cm^{-2})	FF (%)	PCE[a] (%)	PCE_{max} (%)
PBT1-C:	1:1	0.87±0.1	14.3±0.6	69.2±0.3	8.7±0.4	9.2
IDTT-C6-TIC	1:1.3	0.86±0.1	15.9±0.2	68.0±0.6	9.3±0.2	9.5
	1:1.5	0.86±0.1	14.8±0.3	65.6±1.4	8.4±0.1	8.6
PBT1-C:	1:1	0.90±0.1	17.4±0.4	62.2±0.4	9.8±0.2	10.0
IDTT-C8-TIC	1:1.3	0.90±0.1	17.8±0.3	62.0±1.4	9.9±0.3	10.3
	1:1.5	0.89±0.1	17.4±0.2	60.9±0.9	9.4±0.3	9.8
PBT1-C:	1:1	0.94±0.1	15.6±0.4	64.5±0.5	9.5±0.2	9.7
IDTT-C10-TIC	1:1.3	0.93±0.1	16.8±0.3	64.1±0.7	10.0±0.2	10.2
	1:1.5	0.94±0.1	15.6±0.5	64.0±0.6	9.3±0.3	9.7

[a]The average parameters were calculated from 20 independent cells.

Supplementary Table 8. Summary of device parameters of PBT1-C:IDTT-CX-TIC solar cells with different DIO contents under the illumination of AM1.5G, 100 mW cm^{-2}.

Materials	DIO (%)	V_{OC} (V)	J_{SC} (mA cm^{-2})	FF (%)	PCE[a] (%)	PCE_{max} (%)
PBT1-C: IDTT-C6-TIC	0	0.86±0.1	15.9±0.2	68.0±0.6	9.3±0.2	9.5
	0.5	0.85±0.1	17.0±0.3	66.7±2.4	9.6±0.5	10.0
	1	0.86±0.1	14.4±0.3	61.1±2.4	7.5±0.3	7.7
PBT1-C: IDTT-C8-TIC	0	0.90±0.1	17.8±0.3	62.0±1.4	9.9±0.3	10.3
	0.5	0.88±0.1	20.3±0.2	74.6±1.1	13.4±0.2	13.7
	1	0.90±0.1	17.1±0.3	59.0±0.9	9.1±0.1	9.2
PBT1-C: IDTT-C10-TIC	0	0.93±0.1	16.8±0.3	64.1±0.7	10.0±0.2	10.2
	0.5	0.98±0.1	18.1±0.3	71.3±1.3	12.5±0.4	12.7
	1	0.95±0.1	15.9±0.4	57.9±1.1	8.8±0.3	9.1

[a]The average parameters were calculated from 20 independent cells.

Supplementary Table 9. Peak center energy and peak width of the fitting components (Gaussian peaks).

Acceptor	IDTT-C6-TIC	IDTT-C8-TIC	IDTT-C10-TIC
x_{c1}(eV)	1.20± 0.001	1.22 ± 0.001	1.31 ± 0.004
w_1 (eV)	0.065 ± 0.000	0.08 ± 0.001	0.075 ± 0.004
x_{c2} (eV)	1.33 ± 0.000	1.37 ± 0.002	1.45 ± 0.001
w_2 (eV)	0.050 ± 0.000	0.06 ± 0.003	0.05 ± 0.002
x_{c3} (eV)	1.47 ± 0.002	1.47 ± 0.003	1.54 ± 0.002
w_3 (eV)	0.040 ± 0.004	0.03 ± 0.003	0.03 ± 0.002
x_{c4} (eV)	1.55 ± 0.048	1.57 ± 0.001	1.62 ± 0.000
w_4 (eV)	0.072 ± 0.026	0.049 ± 0.001	0.03 ± 0.000

Supplementary Methods

Characterizations of compounds

^{1}H and ^{13}C NMR spectra were recorded on Bruker AV-400/300 (400/300 MHz) NMR spectrometers. Chemical shifts were reported in parts per million (ppm, δ). ^{1}H and ^{13}C NMR spectra were referenced to tetramethylsilane (0 ppm) for $CDCl_3$ as internal standard. Matrix-assisted laser desorption/ionization fourier transform ion cyclotron resonance-mass spectrometry (MALDI-FTICR-MS) was determined on IonSpec 4.7 Tesla Fourier transform mass spectrometer. Molecular weights of the polymers were obtained on a PL GPC 220 (Polymer Laboratories) at 160 °C using a calibration curve of polystyrene standards, with 1,2,4-trichlorobenezene as the eluent.

General information

4,9-dihydro-*s*-indaceno[1,2-*b*:5,6-*b'*]dithieo[3,2-*b*]thiophene was purchased from HYPER Inc. (Zhejiang, China). 2-(6-oxo-5,6-dihydro-4*H*-cyclopenta[*c*]thiophen-4-ylidene)malononitrile was synthesized according to the reported literature[2]. PBT1-C (M_n = 24.5 kDa, M_w = 52.3 kDa, PDI = 2.13) was synthesized according to the reported literature[3]. Other reagents and solvents were purchased from commercial sources and were used without further purification unless stated otherwise. Tetrahydrofuran (THF) was distilled over sodium and benzophenone.

Synthesis and characterization

Synthesis of 6,6,12,12-Tetralkyl-6,12-dihydrodithieno[2,3-d:2',3'-d']-s-indaceno[1,2-b:5,6-b']dithiophene (2).

A solution of 4,9-dihydro-*s*-indaceno[1,2-*b*:5,6-*b'*]dithieo[3,2-*b*]thiophene (0.60 g, 1.59 mmol) and bromoalkane (9.54 mmol , 6 eq) in 50 mL THF was stirred at 0 °C

under N_2. Then *t*-BuOK (1.43 g, 12.72 mmol, 8 eq) was added to the system. The reaction mixture was stirred for 1 hour. 50 mL distilled water was added and the reaction mixture was extracted with dichloromethane (DCM). The organic layer was dried over Na_2SO_4, filtered and concentrated. Then the residue was purified with silica gel chromatography (eluent: petroleum ether) to provide pure product as yellow solid.

Hexyl substitute. (1.02 g, yield: 90.0%).

^{1}H NMR (400 MHz, $CDCl_3$): δ 7.31 (s, 6H), 2.16-2.09 (m, 4H), 2.03-1.96 (m, 4H), 1.14-1.02 (m, 24H), 0.80-0.72 (m, 20H).

^{13}C NMR (101 MHz, $CDCl_3$): δ 152.34, 145.55, 143.76, 141.33, 136.63, 133.70, 125.40, 120.52, 113.02, 54.22, 38.45, 31.49, 29.58, 24.08, 22.52, 13.98.

HRMS (MALDI-FTICR-MS): $[M]^+$ calcd. for $C_{44}H_{58}S_4$, 714.3416; found, 714.3415.

Octyl substitute. (1.15 g, yield: 87.8%).

^{1}H NMR (400 MHz, $CDCl_3$): δ 7.33 (s, 4H), 7.31 (s, 2H), 2.15-2.08 (m, 4H), 2.03-1.95 (m, 4H), 1.14-1.02 (m, 40H), 0.85-0.70 (m, 20H).

^{13}C NMR (101 MHz, $CDCl_3$): δ 151.26, 144.50, 142.68, 140.25, 135.53, 132.62, 124.34, 119.48, 111.96, 53.15, 37.37, 30.75, 28.85, 28.17, 28.14, 23.04, 21.56, 13.03.

HRMS (MALDI-FTICR-MS): $[M]^+$ calcd. for $C_{52}H_{74}S_4$, 826.4668; found, 826.4671.

Decyl substitute. (1.25 g, yield: 83.9%).

^{1}H NMR (400 MHz, $CDCl_3$): δ 7.33-7.30 (m, 6H), 2.16-2.08 (m, 4H), 2.03-1.95 (m, 4H), 1.20-1.07 (m, 56H), 0.86-0.77 (m, 20H).

^{13}C NMR (101 MHz, $CDCl_3$): δ 152.35, 145.56, 143.74, 141.32, 136.60, 133.70, 125.36, 120.49, 113.01, 54.21, 38.39, 31.90, 29.88, 29.55, 29.53, 29.30, 29.25, 24.10, 22.65, 14.08.

HRMS (MALDI-FTICR-MS): $[M]^+$ calcd. for $C_{60}H_{90}S_4$, 938.5920; found, 938.5921.

Synthesis of 6,6,12,12-Tetralkyl-6,12-dihydrodithieno[2,3-d:2',3'-d']-s-indaceno[1,2-b:5,6-b']dithiophene-2,8-dicarboxaldehyde (3).

A solution of compound 2 (0.19 mmol, 1 eq) in 10 mL anhydrous N,N-dimethylformamide (DMF) was stirred at 0 °C under N_2. $POCl_3$ (0.36 mL, 3.87 mmol, 20 eq) was added to the system. The reaction solution was stirred at 80 °C for 8 hours. After cooled at room temperature, the solution was dealt with 20 mL of saturated KOAc solution for 2 hours. Then the reaction mixture was extracted with DCM. The organic layer was dried over Na_2SO_4, filtered and concentrated. Then the residue was purified with silica gel chromatography (eluent: petroleum ether/DCM = 2:1) to provide pure product as yellow solid.

Hexyl substitute. (0.13 g, yield: 86.7%).

^{1}H NMR (400 MHz, $CDCl_3$): δ 9.97 (s, 2H), 8.02 (s, 2H), 7.44 (s, 2H), 2.20-2.13 (m, 4H), 2.07-2.00 (m, 4H), 1.12-1.01 (m, 24H), 0.79-0.68 (m, 20H).

^{13}C NMR (101 MHz, $CDCl_3$): δ 182.86, 153.79, 150.35, 146.10, 143.81, 141.62, 140.04, 137.04, 130.26, 114.19, 54.57, 38.25, 31.42, 29.45, 24.11, 22.48, 13.94.

HRMS (MALDI-FTICR-MS): $[M]^+$ calcd. for $C_{46}H_{58}O_2S_4$, 770.3314; found, 770.3313.

Octyl substitute. (0.15 g, yield: 88.2%).

^{1}H NMR (400 MHz, $CDCl_3$): δ 9.97 (s, 2H), 8.02 (s, 2H), 7.43 (s, 2H), 2.20-2.12 (m, 4H), 2.07-2.01 (m, 4H), 1.15-0.98 (m, 40H), 0.82-0.70 (m, 20H).

^{13}C NMR (101 MHz, $CDCl_3$): δ 182.83, 153.78, 150.35, 146.11, 143.82, 141.61, 140.04, 137.02, 130.23, 114.19, 54.57, 38.22, 31.72, 29.74, 29.14, 29.11, 24.11, 22.57, 14.04.

HRMS (MALDI-FTICR-MS): $[M]^+$ calcd. for $C_{54}H_{74}O_2S_4$, 882.4566; found, 882.4564.

Decyl substitute. (0.17 g, yield: 89.5%).

^{1}H NMR (400 MHz, $CDCl_3$): δ 9.97 (s, 2H), 8.02 (s, 2H), 7.43 (s, 2H), 2.20-2.12 (m, 4H), 2.07-2.00 (m, 4H), 1.20-1.00 (m, 56H), 0.85-0.73 (m, 20H).

^{13}C NMR (101 MHz, $CDCl_3$): δ 182.82, 153.79, 150.34, 146.11, 143.81, 141.61, 140.05, 137.02, 130.20, 114.20, 54.57, 38.20, 31.85, 29.75, 29.50, 29.48, 29.27, 29.19, 24.12, 22.63, 14.08.

HRMS (MALDI-FTICR-MS): $[M]^+$ calcd. for $C_{62}H_{90}O_2S_4$, 994.5818; found, 994.5814.

Synthesis of IDTT-CX-TIC.

A solution of compound 3 (0.13 mmol, 1 eq), 2-(6-oxo-5,6-dihydro-4*H*-cyclopenta[*c*]thiophen-4-ylidene)malononitrile (0.08 g, 0.40 mmol, 3 eq) in 20 mL chloroform was stirred at room temperature. Pyridine (five drops) was added to the system. The reaction solution was stirred at 60 °C overnight. After the solvent was evaporated, the residue was purified with silica gel chromatography (eluent: petroleum ether/DCM = 3:2) to provide pure product as metallic yellowish green solid.

Hexyl substitute. (0.12 g, yield: 80.0%).

^{1}H NMR (300 MHz, $CDCl_3$): δ 8.92 (s, 2H), 8.41 (d, J = 2.2 Hz, 2H), 8.20 (s, 2H), 7.97 (d, J = 2.2 Hz, 2H), 7.49 (s, 2H), 2.31-2.20 (m, 4H), 2.15-2.03 (m, 4H), 1.13-0.97 (m, 24H), 0.78-0.66 (m, 20H).

^{13}C NMR (75 MHz, $CDCl_3$): δ 180.52, 155.18, 153.94, 153.25, 146.57, 146.25, 142.70, 141.57, 141.23, 138.61, 138.13, 136.87, 136.54, 127.36, 126.64, 124.46, 114.07, 113.82, 113.37, 66.50, 53.72, 52.41, 37.27, 30.43, 28.40, 23.12, 21.45, 12.92.

HRMS (MALDI-FTICR-MS): $[M]^+$ calcd. for $C_{66}H_{62}N_4O_2S_6$, 1134.3192; found, 1134.3188.

Octyl substitute. (0.12 g, yield: 75.0%).

^{1}H NMR (300 MHz, $CDCl_3$): δ 8.92 (s, 2H), 8.41 (d, J = 2.2 Hz, 2H), 8.20 (s, 2H), 7.97 (d, J = 2.2 Hz, 2H), 7.49 (s, 2H), 2.30-2.20 (m, 4H), 2.15-2.05 (m, 4H), 1.15-0.98 (m, 40H), 0.82-0.71 (m, 20H).

^{13}C NMR (75 MHz, $CDCl_3$): δ 181.52, 156.19, 154.98, 154.29, 147.63, 147.30, 143.72, 142.59, 142.26, 139.64, 139.17, 137.89, 137.56, 128.37, 127.67, 125.47, 115.11, 114.86, 114.39, 67.52, 54.75, 53.44, 38.25, 31.75, 29.71, 29.18, 29.11, 24.14, 22.58, 14.06.

HRMS (MALDI-FTICR-MS): $[M]^+$ calcd. for $C_{74}H_{78}N_4O_2S_6$, 1246.4444; found, 1246.4432.

Decyl substitute. (0.11 g, yield: 61.1%).

^{1}H NMR (300 MHz, $CDCl_3$): δ 8.92 (s, 2H), 8.41 (d, J = 2.2 Hz, 2H), 8.19 (s, 2H), 7.97 (d, J = 2.2 Hz, 2H), 7.48 (s, 2H), 2.30-2.20 (m, 4H), 2.14-2.04 (m, 4H), 1.17-1.01 (m, 56H), 0.83-0.73 (m, 20H).

^{13}C NMR (101 MHz, $CDCl_3$): δ 181.54, 156.21, 154.98, 154.28, 147.64, 147.30, 143.71, 142.59, 142.27, 139.65, 139.17, 137.88, 137.55, 128.37, 127.67, 125.47, 115.10, 114.85, 114.39, 67.51, 54.75, 38.22, 31.88, 29.69, 29.52, 29.47, 29.35, 29.29, 29.20, 24.12, 22.63, 14.07.

HRMS (MALDI-FTICR-MS): $[M]^+$ calcd. for $C_{82}H_{94}N_4O_2S_6$, 1358.5696; found, 1358.5682.

Single Crystal Growth and Characterization: The single crystals suitable for X-ray diffraction measurement were grown by the liquid diffusion method. A moderate amount of ethanol was transferred into the concentrated toluene solution and the crystals were formed onto the inner glassy tube over time. The X-ray crystallographic data were recorded at around 100 K on Rigaku XtaLAB P2000 FR-X with a rotating copper anode and a Pilatus 200K detector. The structure was solved by intrinsic phasing method (SHELXT) and refined by least squares method (SHELXL) integrated in Olex2.

Supplementary Note 1

Molecular Dynamics (MD) Simulations: All atomistic MD simulations were based on the general AMBER force field[4] and carried out with the Gromacs-4.6.7[5] software package. The IDTT-CX-TIC crystal based on a supercell (6×6×4 for IDTT-C6-TIC; 4×4×3 for IDTT-C8-TIC; 6×3×3 for IDTT-C10-TIC) was equilibrated at 300 K for 5 ns.

Electronic-Structure Calculations: The electronic couplings for molecular pairs in crystals (the shortest interatomic distance between backbones is less than 0.5 nm) were computed using the Zerner's intermediate neglect of differential overlap (ZINDO) method[6].

Electron Mobility Calculations: Following the previous work[7-10], we employed the Kinetic Monte Carlo simulations in combination with the semi-classical Marcus theory to calculate the electron mobilities of the crystals[11,12]. The internal organization energy for IDTT-CX-TIC was estimated by DFT at the B3LYP/6-31G** level (~ 0.155 eV), while the outer organization energy was set as 0.1 eV, a typical value for low dielectric constant mediums according to classical continuum model.[13] Due to the total organization energy (~ 0.255 eV) much larger than the electronic couplings, the Marcus hopping regime should be appropriate for estimating the electron mobilities. The last 50 ps of equilibration was sampled every 5 ps to generate 10 snapshots to consider the thermal fluctuation effect. It should be noted that the contributions from static and dynamic disorders were both included and were not further distinguished.

Supplementary References

1. Vandewal, K. Interfacial Charge Transfer States in Condensed Phase Systems, *Annu. Rev. Phys. Chem.* **67**, 113–133 (2016).
2. Xie, D. *et al.* A Novel Thiophene-Fused Ending Group Enabling an Excellent Small Molecule Acceptor for High-Performance Fullerene-Free Polymer Solar Cells with 11.8% Efficiency. *Solar RRL* **1**, 1700044 (2017).
3. Liu, T. *et al.* Optimized Fibril Network Morphology by Precise Side-Chain Engineering to Achieve High-Performance Bulk-Heterojunction Organic Solar Cells. *Adv. Mater.* **30**, 1707353 (2018).
4. Wang, J. M., Wolf, R. M., Caldwell, J. W., Kollman, P. A. & Case, D. A. Development and testing of a general amber force field. *J. Comput. Chem.* **25**, 1157-1174 (2004).
5. Hess, B., Kutzner, C., van der Spoel, D. & Lindahl, E. GROMACS 4: Algorithms for Highly Efficient, Load-Balanced, and Scalable Molecular Simulation. *J. Chem. Theory Comput.* **4**, 435-447 (2008).
6. Zerner, J. R. a. M. An Intermediate Neglect of Differential Overlap Technique for Spectroscopy: Pyrrole and the Azines. *Theoret. Chim. Acta* **32**, 111-134 (1973).
7. Han, G., Guo, Y., Song, X., Wang, Y. & Yi, Y. Terminal π–π stacking determines three-dimensional molecular packing and isotropic charge transport in an A–π–A electron acceptor for non-fullerene organic solar cells. *J. Mater. Chem. C* **5**, 4852-4857 (2017).
8. Han, G., Guo, Y., Ning, L. & Yi, Y. Improving the Electron Mobility of ITIC by End-Group Modulation: The Role of Fluorination and π-Extension. *Solar RRL* **3**, 1800251 (2019).
9. Han, G., Shen, X., Duan, R., Geng, H. & Yi, Y. Revealing the influence of the solvent evaporation rate and thermal annealing on the molecular packing and charge transport of DPP(TBFu)2. *J. Mater. Chem. C* **4**, 4654-4661 (2016).
10. Guo, Y., Han, G. C., Duan, R. H., Geng, H. & Yi, Y. P. Boosting the electron mobilities of dimeric perylenediimides by simultaneously enhancing intermolecular and intramolecular electronic interactions. *J. Mater. Chem. A* **6**, 14224-14230 (2018).
11. Wang, L. *et al.* Computational methods for design of organic materials with high charge mobility. *Chem. Soc. Rev.* **39**, 423-434 (2010).
12. Marcus, R. A. Electron transfer reactions in chemistry. Theory and experiment. *Rev. Mod. Phys.* **65**, 599-610 (1993).
13. Rühle, V., & Andrienko, D. Microscopic Simulations of Charge Transport in Disordered Organic Semiconductors. *J. Chem. Theory Comput.* **7**, 3335-3345 (2011).
