# Supplementary material for: Unraveling the influence of non-fullerene acceptor molecular packing on photovoltaic performance of organic solar cells
Source: Nat Commun. 2020 Nov 26;11:6005. doi: 10.1038/s41467-020-19853-z (PMC7693324; doi:10.1038/s41467-020-19853-z)
Supplement: Supplementary file 3 — Reporting Summary [file 41467_2020_19853_MOESM3_ESM.pdf]

nature research

Corresponding author(s): Yanming Sun

Solar Cells Reporting Summary

Nature Research wishes to improve the reproducibility of the work that we publish. This form is intended for publication with all accepted papers reporting the characterization of photovoltaic devices and provides structure for consistency and transparency in reporting. Some list items might not apply to an individual manuscript, but all fields must be completed for clarity.

For further information on Nature Research policies, including our data availability policy, see Authors & Referees.

Experimental design

Please check: are the following details reported in the manuscript?

1. Dimensions

Item	Yes	No	Details
Area of the tested solar cells	☒	☐	Section "Device development and testing"
Method used to determine the device area	☒	☐	The electronic active area of the cell is defined by the overlap of the ITO electrode and metal electrode.

2. Current-voltage characterization

Item	Yes	No	Details
Current density-voltage (J-V) plots in both forward and backward direction	☐	☒	Generally, organic photovoltaic devices do not have forward and backward problems. And we only scan the device in forward direction.
Voltage scan conditions<br>*For instance: scan direction, speed, dwell times*	☒	☐	Section "Device development and testing"
Test environment<br>*For instance: characterization temperature, in air or in glove box*	☒	☐	Our devices were characterized at room temperature (ca. 25 Celsius degree) in glove box.
Protocol for preconditioning of the device before its characterization	☐	☒	No preconditioning protocol
Stability of the J-V characteristic<br>*Verified with time evolution of the maximum power point or with the photocurrent at maximum power point; see ref. 7 for details.*	☐	☒	We only tested the long-term stability.

3. Hysteresis or any other unusual behaviour

Item	Yes	No	Details
Description of the unusual behaviour observed during the characterization	☐	☒	No hysteresis or other unusual behaviour was observed during the characterization of the solar cells. In general, organic solar cells do not have hysteresis problems.
Related experimental data	☐	☒	No hysteresis or other unusual behaviour was observed during the characterization of the solar cells.

4. Efficiency

Item	Yes	No	Details
External quantum efficiency (EQE) or incident photons to current efficiency (IPCE)	☒	☐	The external quantum efficiency (EQE) spectra of OSCs are recorded using Enli QE-R3011 (Enli Technology Co., Ltd Taiwan).
A comparison between the integrated response under the standard reference spectrum and the response measure under the simulator	☒	☐	There are less than 5% errors between the integrated response under the standard reference spectrum and the response measure under the simulator. We give details in the manuscript.
For tandem solar cells, the bias illumination and bias voltage used for each subcell	☐	☒	We only fabricated single junction solar cells.

5. Calibration

Item	Yes	No	Details
Light source and reference cell or sensor used for the characterization	☒	☐	Section "Device development and testing"
Confirmation that the reference cell was calibrated and certified	☒	☐	Section "Device development and testing"

Calculation of spectral mismatch between the reference cell and the devices under test	☒ Yes ☐ No	The relative expanded uncertainty resulting of the relative combined standard uncertainty multiplied with a coverage factor k = 2 is specified. It corresponds to a level of confidence of 95%.

6. Mask/aperture

Size of the mask/aperture used during testing	☐ Yes ☒ No	We did not use a mask during testing and the device effective area was obtained by its crossed area of the bottom and top electrodes.
Variation of the measured short-circuit current density with the mask/aperture area	☐ Yes ☒ No	We did not use a mask during testing.

7. Performance certification

Identity of the independent certification laboratory that confirmed the photovoltaic performance	☐ Yes ☒ No	As we focus on studying the influence of NFA molecular packing on photovoltaic performance of OSCs, we have not certified the efficiency values.
A copy of any certificate(s) *Provide in Supplementary Information*	☐ Yes ☒ No	No certification.

8. Statistics

Number of solar cells tested	☒ Yes ☐ No	Number of solar cells tested is provided in Table 1.
Statistical analysis of the device performance	☒ Yes ☐ No	Statistical results of the devices are listed in Table 1.

9. Long-term stability analysis

Type of analysis, bias conditions and environmental conditions *For instance: illumination type, temperature, atmosphere humidity, encapsulation method, preconditioning temperature*	☒ Yes ☐ No	See "Supplementary Fig. 47"
